# Discontinuation of Oral Anticoagulation After Successful Atrial Fibrillation Ablation

**DOI:** 10.1001/jamanetworkopen.2025.1320

**Published:** 2025-03-21

**Authors:** Tomoya Iwawaki, Satoshi Yanagisawa, Yasuya Inden, Kei Hiramatsu, Ryota Yamauchi, Kiichi Miyamae, Hiroyuki Miyazawa, Takayuki Goto, Shun Kondo, Masaya Tachi, Masafumi Shimojo, Yukiomi Tsuji, Toyoaki Murohara

**Affiliations:** 1 Department of Cardiology, Nagoya University Graduate School of Medicine, Nagoya, Japan; 2 Department of Advanced Cardiovascular Therapeutics, Nagoya University Graduate School of Medicine, Nagoya, Japan

## Abstract

**Importance:**

There is no clear consensus regarding the discontinuation of oral anticoagulants (OACs) after catheter ablation (CA) for atrial fibrillation (AF).

**Objective:**

To evaluate thromboembolic and major bleeding events and all-cause death following OAC discontinuation and characteristics associated with patient prognoses after successful CA.

**Design, Setting, and Participants:**

This retrospective cohort study included patients without AF recurrence or adverse events 12 months after CA among those undergoing their first CA between January 1, 2006, and December 31, 2021. The study population was divided into groups according to the continuation and discontinuation of OACs at the landmark period of 12 months after CA. Follow-up data were acquired until December 31, 2023, and the study analysis was conducted from January to April 2024.

**Exposures:**

OAC discontinuation.

**Main Outcomes and Measures:**

Primary outcomes were thromboembolic and major bleeding events and all-cause death after 12 months. Inverse probability of treatment weighting (IPTW) and propensity score–matched analyses were used to adjust baseline characteristics.

**Results:**

This study included 1821 patients (mean [SD] age, 63.6 [11.7] years; 1339 men [73.5%]). Overall, 922 patients (50.6%) continued OAC for 12 months, whereas 899 (49.4%) discontinued OAC. During a mean (SD) follow-up of 4.8 (4.0) years, thromboembolic events, major bleeding events, and death occurred in 43 (2.4%), 41 (2.3%), and 71 (3.9%) patients, respectively. After IPTW adjustment, the OAC discontinuation group demonstrated a significantly higher incidence of thromboembolism (incidence rate, 0.86 [95% CI, 0.45-1.35] vs 0.37 [95% CI, 0.22-0.54] per 100 person-years; log-rank *P* = .04) and a lower incidence of major bleeding (incidence rate, 0.10 [95% CI, 0.02-0.19] vs 0.65 [95% CI, 0.43-0.90] per 100 person-years; log-rank *P* < .001) than in the continuation group. In a subgroup analysis, OAC discontinuation was associated with a higher risk of thromboembolism in patients with asymptomatic AF, left ventricular ejection fraction of less than 60%, and left atrial diameter of 45 mm or greater. In contrast, OAC discontinuation was beneficial for reducing major bleeding risks in patients with a HAS-BLED score of 2 or greater. These outcomes were similar in the propensity score–matched analysis using 1100 paired matched patients, except for insignificant differences in thromboembolic events. Differences in mortality between the 2 groups were not statistically significant.

**Conclusions and Relevance:**

In this retrospective cohort study, discontinuation of OACs after successful CA was associated with increased thromboembolic events and decreased bleeding events. The benefits of discontinuing OACs were stratified according to specific characteristics, pending a future prospective randomized study.

## Key Points

**Question:**

What patient characteristics are associated with benefit following oral anticoagulant (OAC) discontinuation after successful catheter ablation (CA) for atrial fibrillation (AF)?

**Findings:**

In this cohort study of 1821 patients, thromboembolic risk increased in the OAC discontinuation group 12 months after successful CA, while the OAC continuation group exhibited more bleeding events. OAC discontinuation was associated with increased thromboembolic risk in patients with asymptomatic AF and poor cardiac function, whereas it was associated with decreased bleeding risk in patients with a HAS-BLED score of 2 or greater.

**Meaning:**

These findings suggest that a better balance of the risk estimation between 2 conflicting events should be constructed based on the specific characteristics.

## Teaser

This cohort study evaluates the association between oral anticoagulant discontinuation and prognosis after successful catheter ablation for atrial fibrillation.

## Introduction

Catheter ablation (CA) is widely used to effectively suppress the incidence of atrial fibrillation (AF), and recent technological advances have made CA more useful and safer.^1,2,3^ However, because the cumulative rate of recurrence increases even in a limited follow-up examination, postablation continuation of oral anticoagulants (OACs) is recommended for patients with a high thrombotic risk of several comorbidities.^4,5,6,7,8^ In contrast, OAC continuation for no specific reason increases the risk of bleeding events. The balance between risks of thromboembolism and bleeding should be carefully considered when OACs are continued following CA. Notably, previous research evaluating the risks and benefits of OAC discontinuation following CA have been limited to retrospective observational studies, and no prospective randomized study has been reported to date.^4,5,7,9,10,11,12,13,14,15,16^

We hypothesized that several specific characteristics and detailed background characteristics may be associated with thromboembolic and bleeding events individually and that extracting the specific demographic characteristics associated with adverse events may help decide whether to continue or discontinue OACs after successful CA with clinical utility. To explore this hypothesis, we evaluated the risks and benefits associated with OAC discontinuation vs continuation after CA for AF using a large-scale CA database of an institution with more than 15 years of experience with the procedure. We further extracted specific background characteristics associated with adverse events to identify a group of patients who may more safely and effectively benefit from discontinuing or continuing OACs after CA.

## Methods

The Institutional Ethics Review Board of Nagoya University Hospital approved the study protocol and dataset for this cohort study. Written informed consent was obtained from all patients before the CA procedure. This study followed the Strengthening the Reporting of Observational Studies in Epidemiology (STROBE) reporting guideline.

### Study Population

**Figure 1.**
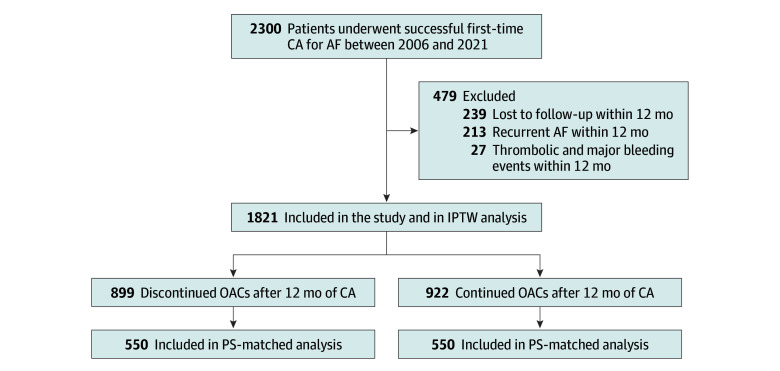
Flowchart of Patients Included in This Study AF indicates atrial fibrillation; CA, catheter ablation; IPTW, inverse probability of treatment weighting; OAC, oral anticoagulant; PS, propensity score.

The study population was retrospectively recruited from an ablation dataset at Nagoya University Hospital. The study initially evaluated 2300 patients who underwent their first CA for AF between January 1, 2006, and December 31, 2021. Follow-up data were acquired until December 31, 2023, and study analyses were performed between January and April 2024. Because of a statistical method using landmark analysis for the follow-up period after 12 months in this study, we excluded patients who were lost to follow-up within 12 months of CA; those who had recurrent AF within 12 months, except for patients who had early recurrence within 3 months (a blanking period) following the procedure; and those who had any thromboembolic and major bleeding events within 12 months of CA (Figure 1). The indications for CA were established per the latest guidelines.^1,17^

### Perioperative Anticoagulation Management and Study Group Design

**Figure 2.**
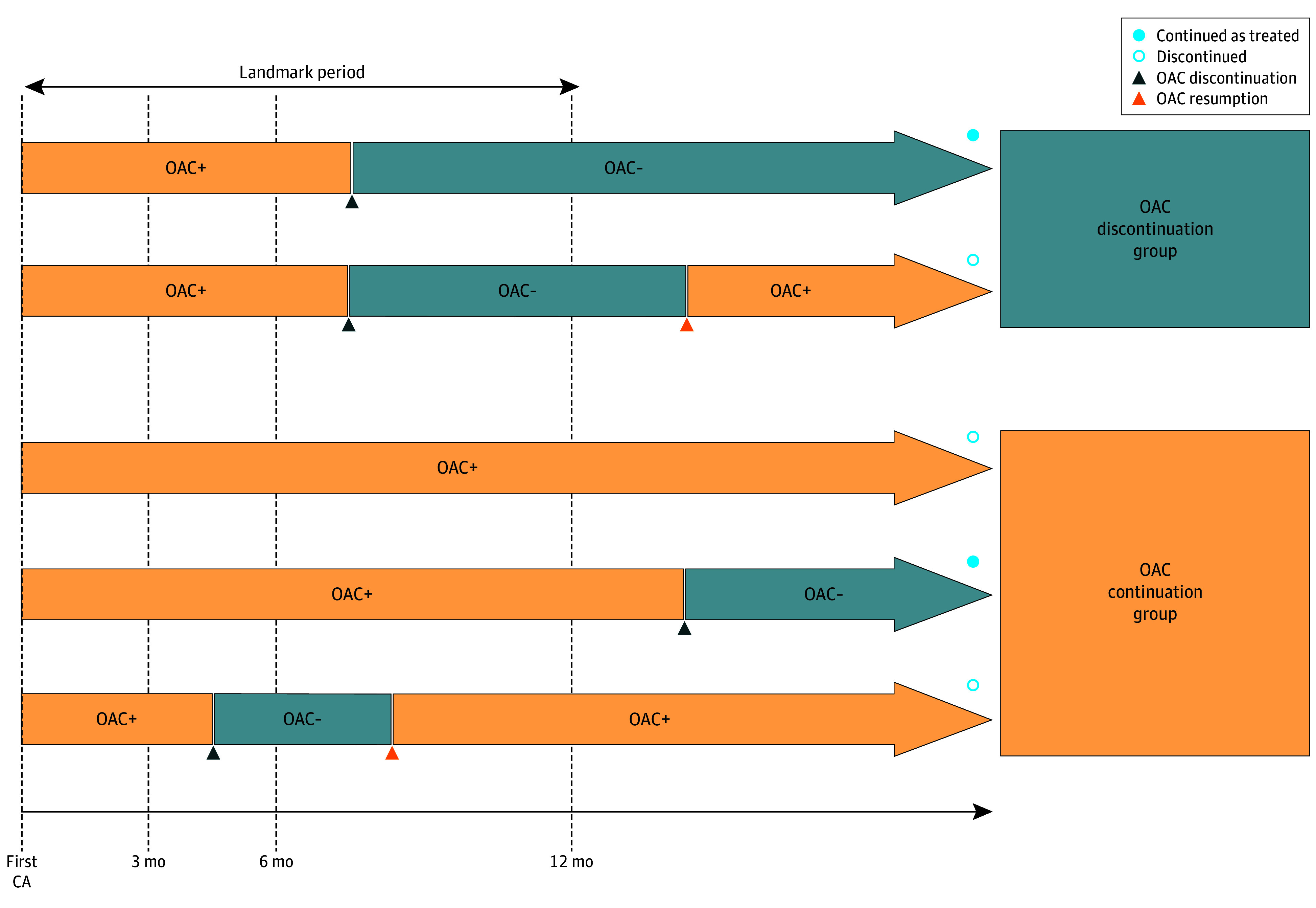
Definitions of Oral Anticoagulant (OAC) Discontinuation and Continuation Groups After Catheter Ablation (CA) for Atrial Fibrillation (AF) A landmark period was set to 12 months after CA.

Patients were administered OACs for at least 3 to 4 weeks before CA. Typical examinations and CA approaches are summarized in the eMethods in Supplement 1.^18,19^ Following CA, OACs were continued for at least 3 months and were further continued or discontinued at the discretion of the attending physician when patients had no evidence of AF recurrence. The study population was categorized into 2 groups: OAC discontinuation and OAC continuation. The landmark period was defined as 12 months after CA. The OAC discontinuation group included patients who discontinued OACs within the landmark period and did not receive OAC medications 12 months after CA. The OAC continuation group included patients who were taking OACs for a landmark period of 12 months. Patients who discontinued OACs at the time of the landmark period but resumed OACs thereafter were assigned to the OAC discontinuation group. In contrast, patients were included in the OAC continuation group if they resumed OACs at 12 months after temporal discontinuation of OACs within the landmark period (Figure 2).

### Follow-Up Assessment and Outcomes

All patients were examined under continuous electrocardiographic (ECG) monitoring for 3 days after the procedure. After discharge, patients were typically scheduled to visit our institution’s outpatient clinic at 1, 3, 6, 9, and 12 months after CA and to visit a general practitioner at a minimum of every month. At each visit, all patients were examined using a 3-minute surface 12-lead electrocardiogram. All patients underwent 24-hour Holter ECG monitoring after 1 month or longer. Additional methods to detect reoccurrence are described in the eMethods in Supplement 1.

The primary outcomes were thromboembolic and major bleeding events, all-cause deaths, and AF recurrence occurring over 12 months after CA (eMethods in Supplement 1). Major bleeding was defined according to the International Society on Thrombosis and Haemostasis definition of bleeding complications.^20^ Thromboembolism included ischemic stroke, systemic embolism, and transient ischemic attack confirmed by clinical and imaging evaluation. AF recurrence was defined as the first AF or atrial tachycardia lasting longer than 30 seconds on examination testing after the blanking period of CA. The primary end point was defined as thromboembolic events in the main analysis and in the as-treated analysis. The aforementioned outcomes were compared between the OAC continuation and discontinuation groups. Subgroup analyses were then performed to evaluate which parameters would benefit from the discontinuation or continuation of OACs for each event.

### Statistical Analysis

Differences in the numeric values of the 2 groups were analyzed using the *t* test for normally distributed data and the Mann-Whitney *U* test for nonnormally distributed data. Categorical variables were analyzed using the Fisher exact test or the χ^2^ test.

To stabilize the baseline characteristics between the OAC continuation and discontinuation groups, an inverse probability of treatment weighting (IPTW) analysis was performed to weigh each individual propensity score (PS) via the inverse probability of receiving either treatment. Details are provided in the eMethods in Supplement 1. Moreover, we performed PS-matched analysis and an alternative analysis through an as-treated approach regarding the follow-up period, which was defined as the OAC medication strategy at the last follow-up time or the time of the adverse event, to assess the outcomes from the different viewpoints and aspects. In the PS-matched analysis, 1:1 nearest-neighbor greedy matching was performed based on the calculated score individually. Finally, an additional IPTW analysis was performed according to the landmark period of 6 months after CA. Subgroup analysis of outcomes was performed using Cox proportional hazards regression analysis and interaction tests. Statistical significance was set at *P* < .05 (2-tailed). Data were analyzed with SPSS, version 28.0 (SPSS Inc); and R, version 4.3.3 (R Project for Statistical Computing).

## Results

### Baseline Characteristics of the OAC Discontinuation and Continuation Groups

This study included 1821 patients; 899 (49.4%) and 922 (50.6%) were assigned to the OAC discontinuation and continuation groups, respectively (Figure 1 and Table). Their mean (SD) age was 63.6 (11.7) years; there were 1339 men (73.5%) and 482 women (26.5%). Considerable differences between the 2 groups were observed for most parameters. For example, the OAC discontinuation group was younger and included more male patients than the continuation group. The OAC discontinuation group also had a higher prevalence of paroxysmal AF and symptomatic AF and lower CHADS_2_ (congestive heart failure, hypertension, age, diabetes, and stroke), CHA_2_DS_2_-VASc (congestive heart failure, hypertension, aged ≥75 years, diabetes, stroke–vascular disease, aged 65-74 years, and female sex), and HAS-BLED (hypertension, kidney or liver disease, stroke history, prior bleeding, unstable international normalized ratio, aged >65 years, and drug or alcohol use) scores than the continuation group. Conversely, the OAC continuation group had considerably higher rates of warfarin use, antiplatelet drug use, and medications for heart failure than the OAC discontinuation group.

**Table.** Baseline Characteristics of the OAC Discontinuation and Continuation Groups^a^

Characteristic	OAC discontinuation (n = 899)	OAC continuation (n = 922)	*P* value (before adjustment)	SMD (95% CI)	
				Before adjustment	After adjustment
Age, mean (SD), y	60.5 (12.1)	66.6 (10.4)	<.001	0.54 (0.45-0.63)	0.02 (−0.07 to 0.11)
Sex					
Male	696 (77.4)	643 (69.7)	<.001	0.18 (0.13-0.22)	0.03 (−0.06 to 0.12)
Female	203 (22.6)	279 (30.3)	<.001	0.18 (0.13-0.22)	0.03 (−0.06 to 0.12)
BMI, mean (SD)	23.9 (3.5)	24.2 (3.9)	.17	0.08 (−0.01 to 0.17)	0.03 (−0.06 to 0.12)
AF duration, mean (SD), y	2.6 (3.6)	2.9 (4.4)	.13	0.08 (−0.01 to 0.17)	0.05 (−0.04 to 0.14)
AF type					
Paroxysmal	644 (71.6)	555 (60.2)	<.001	0.25 (0.20-0.29)	0.01 (−0.08 to 0.10)
Nonparoxysmal	255 (28.4)	367 (39.8)	<.001	0.25 (0.20-0.29)	0.01 (−0.08 to 0.10)
Symptomatic	655 (72.9)	584 (63.3)	<.001	0.21 (0.16-0.25)	0.01 (−0.08 to 0.10)
Hemodialysis	3 (0.3)	4 (0.4)	>.99	0.02 (−0.03 to 0.06)	0.01 (−0.08 to 0.10)
Thrombosis score, mean (SD)					
CHADS2 score	0.71 (0.84)	1.51 (1.12)	<.001	0.81 (0.71-0.90)	0.03 (−0.06 to 0.12)
CHA2DS2-VASc score	1.43 (1.32)	2.56 (1.57)	<.001	0.77 (0.68-0.87)	0.04 (−0.05 to 0.13)
Bleeding score (HAS-BLED), mean (SD)	1.03 (0.97)	1.65 (1.05)	<.001	0.61 (0.52-0.70)	0.03 (−0.06 to 0.12)
Laboratory data					
BNP level, median (IQR), pg/mL	37.9 (16.8–87.6)	74.8 (34.8–149.9)	<.001	0.28 (0.19-0.37)	0.04 (−0.05 to 0.13)
eGFR, mean (SD), mL/min/1.73 m2	72.3 (21.2)	64.3 (18.4)	<.001	0.41 (0.32-0.50)	0.14 (0.05-0.23)
PT-INR, mean (SD)	1.46 (0.57)	1.53 (0.56)	.001	0.14 (0.05-0.23)	0.01 (−0.08 to 0.10)
APTT, mean (SD), s	40.9 (9.9)	41.1 (9.5)	.71	0.02 (−0.07 to 0.11)	0.05 (−0.04 to 0.14)
D-dimer, mean (SD), μg/mL	0.67 (1.71)	0.79 (2.18)	<.001	0.06 (−0.03 to 0.15)	<0.01 (−0.08 to 0.10)
Echocardiographic data					
LVEF, mean (SD), %	62.4 (8.2)	59.5 (11.1)	<.001	0.30 (0.21-0.39)	<0.01 (−0.08 to 0.10)
LAD, mean (SD), mm	37.9 (6.1)	41.1 (7.3)	<.001	0.47 (0.38-0.56)	0.02 (−0.07 to 0.11)
MR (moderate or greater)	13 (1.4)	47 (5.1)	<.001	0.21 (0.12-0.30)	0.09 (0-0.18)
TR (moderate or greater)	15 (1.7)	39 (4.2)	.001	0.15 (0.06-0.24)	0.04 (−0.05 to 0.13)
Comorbidity					
Hypertension	356 (39.6)	541 (58.7)	<.001	0.39 (0.34-0.43)	0.03 (−0.06 to 0.12)
Diabetes	89 (9.9)	189 (20.5)	<.001	0.30 (0.25-0.35)	0.05 (−0.04 to 0.14)
Heart failure	63 (7.0)	228 (24.7)	<.001	0.50 (0.41-0.59)	<0.01 (−0.08 to 0.10)
Ischemic heart disease	51 (5.7)	85 (9.2)	.004	0.14 (0.11-0.17)	0.04 (−0.05 to 0.13)
Ischemic stroke	23 (2.6)	97 (10.5)	<.001	0.33 (0.30-0.36)	0.02 (−0.07 to 0.11)
Vascular disease	59 (6.6)	96 (10.4)	.003	0.14 (0.11-0.17)	<0.01 (−0.08 to 0.10)
Device implantation					
Pacemaker	4 (0.4)	24 (2.6)	<.001	0.18 (0.15-0.21)	0.03 (−0.06 to 0.12)
ICD	2 (0.2)	20 (2.2)	<.001	0.18 (0.15-0.21)	0.11 (0.02-0.20)
CRT	2 (0.2)	11 (1.2)	.01	0.12 (0.08-0.17)	0.12 (0.03-0.21)
Ablation procedure					
PV isolation	899 (100)	922 (100)	>.99	NA	NA
CTI ablation	613 (68.2)	681 (73.9)	.008	0.13 (0.04-0.22)	0.02 (−0.07 to 0.11)
Bottom line ablation	159 (17.7)	222 (24.1)	.001	0.16 (0.12-0.19)	0.01 (−0.08 to 0.10)
Roof line ablation	214 (23.8)	299 (32.4)	<.001	0.20 (0.11-0.29)	0.05 (−0.04 to 0.14)
SVC isolation	35 (3.9)	49 (5.3)	.15	0.07 (−0.02 to 0.16)	0.05 (−0.04 to 0.14)
MI ablation	115 (12.8)	176 (19.1)	<.001	0.17 (0.08-0.26)	0.09 (0.06-0.12)
Radiofrequency ablation	679 (75.5)	760 (82.4)	<.001	0.17 (0.08-0.26)	0.04 (−0.05 to 0.13)
Cryoballoon ablation	190 (21.1)	133 (14.4)	<.001	0.18 (0.09-0.27)	<0.01 (−0.08 to 0.10)
Hot balloon ablation	25 (2.8)	23 (2.5)	.70	0.02 (−0.07 to 0.11)	0.06 (−0.03 to 0.15)
Laser balloon ablation	5 (0.6)	6 (0.7)	.80	0.01 (−0.08 to 0.10)	0.08 (−0.01 to 0.18)
Medication					
Warfarin	294 (32.7)	357 (38.7)	.007	0.13 (0.04-0.22)	<0.01 (−0.08 to 0.10)
DOAC	605 (67.3)	565 (61.3)	.007	0.13 (0.04-0.22)	<0.01 (−0.08 to 0.10)
Antiplatelet drug	72 (8.0)	121 (13.1)	<.001	0.17 (0.08-0.26)	0.04 (−0.05 to 0.13)
ACEI or ARB	243 (27.0)	411 (44.6)	<.001	0.37 (0.28-0.46)	0.06 (−0.03 to 0.15)
β-Blocker	322 (35.8)	455 (49.3)	<.001	0.28 (0.20-0.37)	0.09 (0-0.18)
Loop diuretic	55 (6.1)	181 (19.6)	<.001	0.41 (0.32-0.50)	0.03 (−0.06 to 0.12)
MRA	26 (2.9)	115 (12.5)	<.001	0.37 (0.34-0.40)	0.08 (−0.01 to 0.17)
AAD (I)	329 (36.6)	290 (31.5)	.02	0.25 (0.16-0.34)	0.02 (−0.07 to 0.11)
AAD (III)	75 (8.3)	150 (16.3)	<.001	0.25 (0.16-0.34)	0.02 (−0.07 to 0.11)

Abbreviations: AAD, antiarrhythmic drug; ACEI, angiotensin-converting enzyme inhibitor; AF, atrial fibrillation; APTT, activated partial thromboplastin time; ARB, angiotensin II receptor blocker; BMI, body mass index (calculated as weight in kilograms divided by height in meters squared); BNP, brain natriuretic peptide; CHADS_2_, congestive heart failure, hypertension, age, diabetes, and stroke; CHA_2_DS_2_-VASc, congestive heart failure, hypertension, aged 75 years or older, diabetes, stroke–vascular disease, aged 65 to 74 years, and female sex; CRT, cardiac resynchronization therapy; CTI, cavo tricuspid isthmus; DOAC, direct oral anticoagulant; eGFR, estimated glomerular filtration rate; HAS-BLED, hypertension, kidney or liver disease, stroke history, prior bleeding, unstable international normalized ratio, aged older than 65 years, and drug or alcohol use; ICD, implantable cardioverter defibrillator; LAD, left atrial diameter; LVEF, left ventricular ejection fraction; MI, mitral isthmus; MR, mitral valve regurgitation; MRA, mineralocorticoid receptor antagonist; NA, not applicable; OAC, oral anticoagulant; PT-INR, prothrombin time-international normalized ratio; PV, pulmonary vein; SMD, standardized mean difference; SVC, superior vena cava; TR, tricuspid valve regurgitation.

^a^ Unless indicated otherwise, values are presented as No. (%) of patients.

### IPTW Analysis and Prognoses

**Figure 3.**
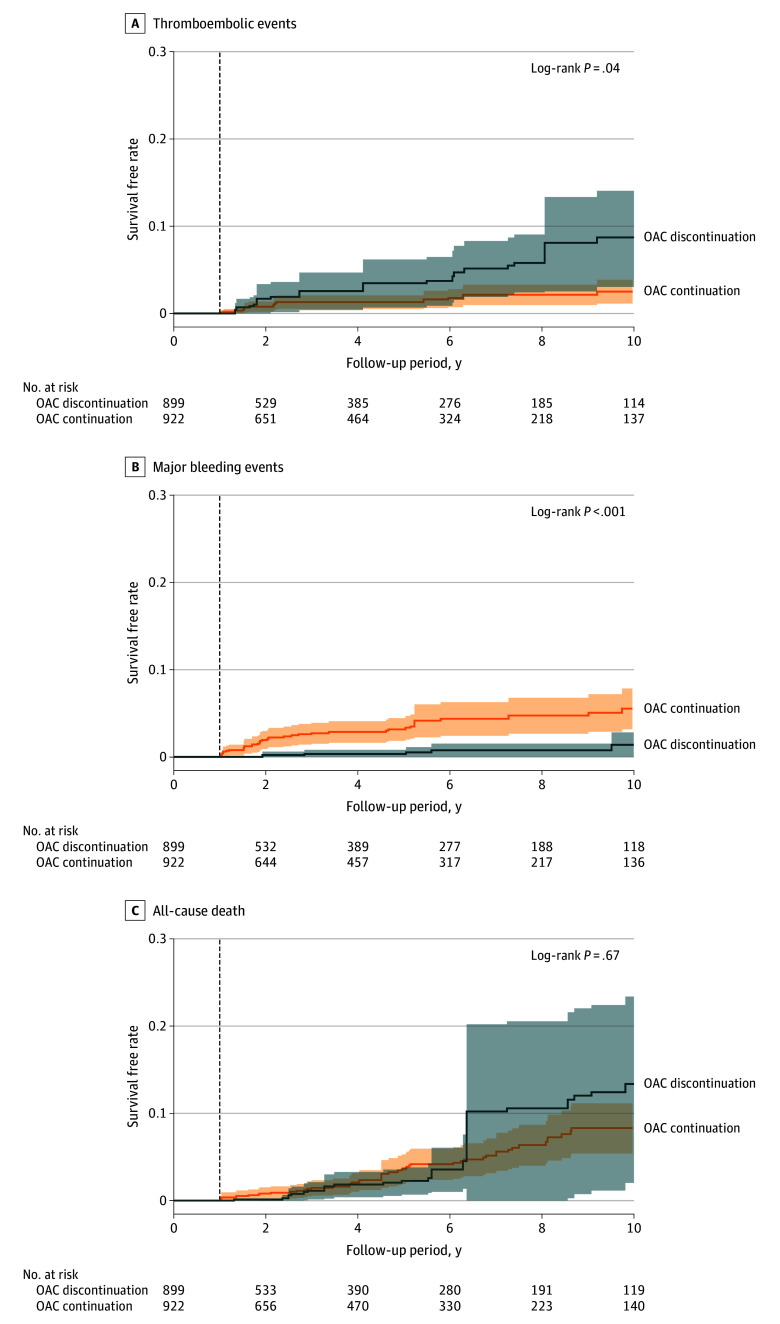
Outcomes After Inverse Probability of Treatment Weighting (IPTW) Analysis A to C, Kaplan-Meier curve analysis of thromboembolic events (A), major bleeding events (B), and all-cause death (C) between the oral anticoagulant (OAC) discontinuation and continuation groups after IPTW analysis.

Thromboembolic events, major bleeding events, and death occurred in 43 (2.4%), 41 (2.3%), and 71 (3.9%) patients, respectively. After PS adjustment using IPTW analysis, distributions of weighted PSs were well balanced between the 2 groups (eFigures 1 and 2 in Supplement 1). During a mean (SD) follow-up of 4.8 (4.0) years, Kaplan-Meier survival analysis with weighted cumulative incidence curves after IPTW demonstrated that the incidence of thromboembolic events was significantly higher in the OAC discontinuation group than in the OAC continuation group (incidence rate, 0.86 [95% CI, 0.45-1.35] vs 0.37 [95% CI, 0.22-0.54] per 100 person-years; log-rank *P* = .04) (Figure 3A). Conversely, the incidence of major bleeding events was significantly higher in the OAC continuation group than in the discontinuation group (incidence rate, 0.65 [95% CI, 0.43-0.90] vs 0.10 [95% CI, 0.02-0.19] per 100 person-years; log-rank *P* < .001) (Figure 3B). The incidence of all-cause death was not statistically significantly different between the OAC continuation and discontinuation groups (incidence rate, 0.99 [95% CI, 0.40-1.88] vs 0.82 [95% CI, 0.58-1.09] per 100 person-years; log-rank *P* = .67) (Figure 3C). The details of each event, annual incidence rates of outcomes, and absolute and relative risk differences are presented in eTables 1, 2, and 3 in Supplement 1, respectively.

Actual OAC administration at the time of adverse events is summarized in eTable 4 in Supplement 1. Most patients continued the assigned OAC regimen, and a few shifted to the other group. Although the incidence of AF after 12 months was significantly lower in the OAC discontinuation group than in the OAC continuation group (155 [17.2%] vs 194 [21.0%]; *P* = .04), the recurrence rates at the time of the events were not significantly different between groups (eTable 4 in Supplement 1).

### Subgroup Analysis Across the Baseline Characteristics

**Figure 4.**
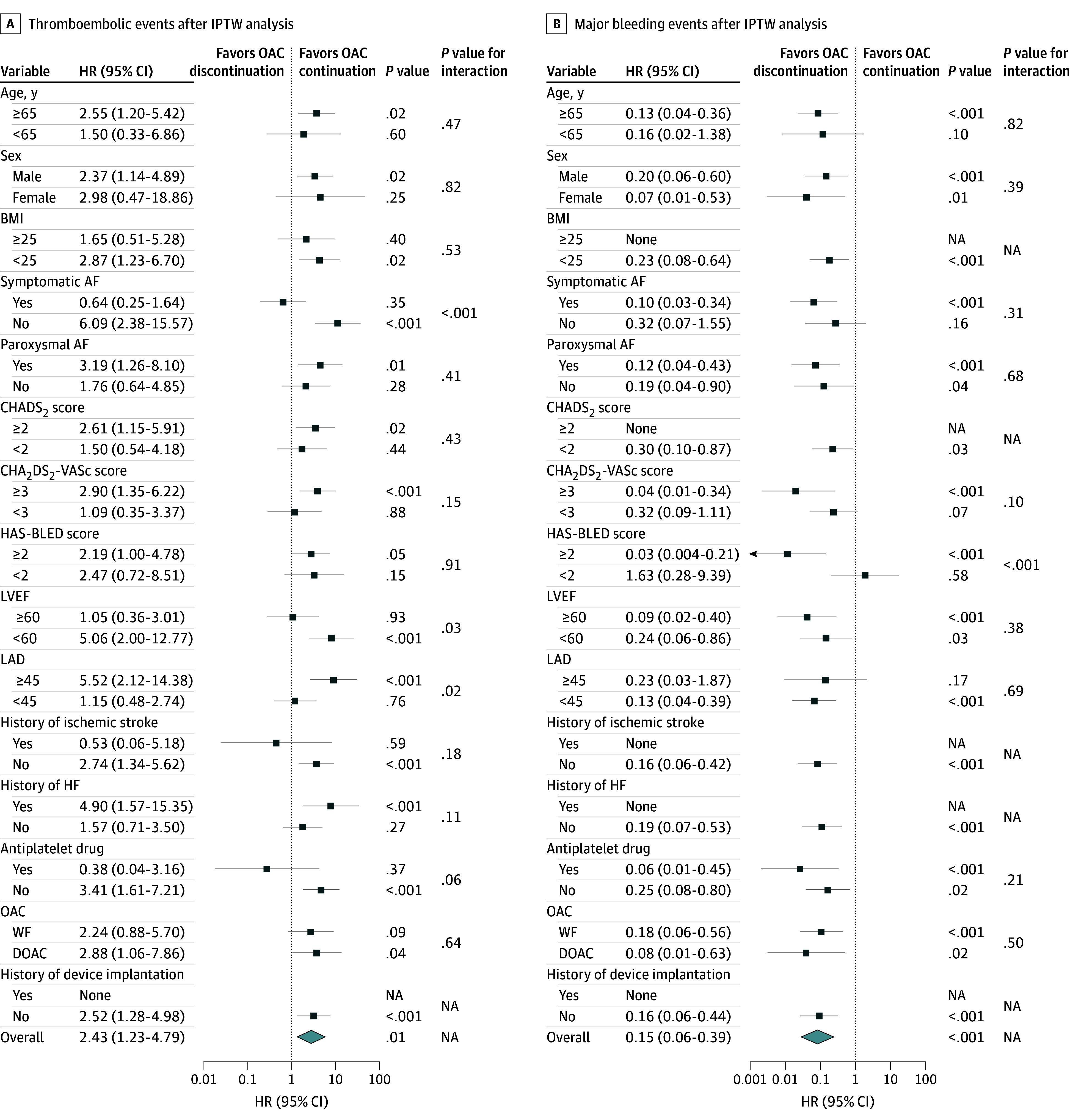
Subanalysis of Outcomes After Inverse Probability of Treatment Weighting (IPTW) Analysis A and B, Subgroup analysis of thromboembolic events (A) and major bleeding events (B) between the oral anticoagulant (OAC) discontinuation and continuation groups after IPTW analysis. OAC continuation was used as a reference. AF indicates atrial fibrillation; BMI, body mass index (calculated as weight in kilograms divided by height in meters squared); CHADS_2_, congestive heart failure, hypertension, age, diabetes, and stroke; CHA_2_DS_2_-VASc, congestive heart failure, hypertension, aged 75 years or older, diabetes, stroke–vascular disease, aged 65 to 74 years, and female sex; DOAC, direct oral anticoagulant; HAS-BLED, hypertension, kidney or liver disease, stroke history, prior bleeding, unstable international normalized ratio, aged older than 65 years, and drug or alcohol use; HF, heart failure; HR, hazard ratio; LAD, left atrial diameter; LVEF, left ventricular ejection fraction; NA, not applicable; WF, warfarin.

Subgroup analysis of thromboembolic events after IPTW showed a significant difference in the interaction between symptomatic and asymptomatic AF (hazard ratio [HR], 0.64 [95% CI, 0.25-1.64] vs 6.09 [95% CI, 2.38-15.57]; interaction *P* < .001), left ventricular ejection fraction (LVEF) (≥60% vs <60%: HR, 1.05 [95% CI, 0.36-3.01] vs 5.06 [95% CI, 2.00-12.77]; interaction *P* = .03), and left atrial diameter (LAD) (≥45 mm vs <45 mm: HR, 5.52 [95% CI, 2.12-14.38] vs 1.15 [95% CI, 0.48-2.74]; interaction *P* = .02) (Figure 4A). Namely, there was a higher risk of thromboembolic events in the OAC discontinuation group among patients with asymptomatic AF, LVEF (<60%), and LAD (≥45 mm) compared with those in the OAC continuation group. In most subgroups, the incidence of major bleeding events was higher in the OAC continuation group. A significant benefit of OAC discontinuation was observed in patients with a HAS-BLED score of 2 or greater compared with those with a score of less than 2 (HR, 0.03 [95% CI, 0.004-0.21] vs 1.63 [95% CI, 0.28-9.39]; interaction *P* < .001) (Figure 4B). Although there was no notable difference in the overall risk of mortality between the 2 groups, there was a significant interaction effect between symptomatic and asymptomatic AF (HR, 0.52 [95% CI, 0.24-1.13] vs 3.27 [95% CI, 1.13-9.44]; interaction *P* < .001) and between patients with and without a history of device implantation (HR, 2.76 [95% CI, 0.92-8.27] vs 0.81 [95% CI, 0.44-1.50]; interaction *P* = .02) (eFigure 4 in Supplement 1).

### Prognosis in the PS-Matched Analysis

Among the study population, 1100 matched patients were extracted after PS-matched analysis. The baseline characteristics of the 2 groups after PS-matched analysis were similar, except for AF duration and prevalence of implantable cardioverter defibrillators, mineralocorticoid receptor antagonists, and class III antiarrhythmic drugs (eTable 5 in Supplement 1). Kaplan-Meier survival curve analysis demonstrated no significant difference in thromboembolic events between the 2 groups (incidence rate, 0.39 [95% CI, 0.16-0.62] vs 0.61 [95% CI, 0.32-0.91] per 100 person-years; log-rank *P* = .24) (eFigure 3A in Supplement 1), whereas the incidence of major bleeding events was significantly higher in the OAC continuation group than in the OAC discontinuation group (incidence rate, 0.61 [95% CI, 0.33-0.90] vs 0.19 [95% CI, 0.02-0.35] per 100 person-years; log-rank *P* = .02) (eFigure 3B in Supplement 1). The incidence of all-cause death was not significantly different between the 2 groups (incidence rate, 0.97 [95% CI, 0.63-1.31] vs 0.62 [95% CI, 0.29-0.94] per 100 person-years; log-rank *P* = .30) (eFigure 3C in Supplement 1). eTable 6 in Supplement 1 lists the detailed events in the population after PS-matched analysis.

In the subgroup analysis of thromboembolic events, a significant interaction was observed between symptomatic and asymptomatic AF (HR, 0.65 [95% CI, 0.21-1.97] vs 4.37 [95% CI, 1.22-15.66]; interaction *P* = .03), LVEF (≥60% vs <60%: HR, 0.63 [95% CI, 0.21-1.93] vs 4.43 [1.23-15.90]; interaction *P* = .02), and LAD (≥45 mm vs <45 mm: HR, 5.70 [95% CI, 1.24-26.07] vs 0.69 [95% CI, 0.25-1.95]; interaction *P* = .02) (eFigure 5A in Supplement 1) in the OAC continuation group. There was also a significant interaction of the major bleeding events between HAS-BLED scores (≥2 vs <2: HR, 0.06 [95% CI, 0.008-0.46] vs 2.44 [95% CI, 0.45-13.32]; interaction *P* = .006) (eFigure 5B in Supplement 1). Additionally, a significant interaction of all-cause death was observed between patients with and without a history of heart failure (HR, 0.23 [95% CI, 0.06-0.84] vs 1.11 [95% CI, 0.54-2.27]; interaction *P* = .02) in the OAC continuation group (eFigure 5C in Supplement 1).

### Prognosis According to As-Treated Analysis and IPTW Analysis at 6-Month Time Point

In the as-treated analysis, 905 and 916 patients were assigned to the OAC and non-OAC groups, respectively. During a median follow-up period of 3.5 years (IQR, 1.2-7.2 years), Kaplan-Meier survival analysis with a weighted cumulative incidence curve after IPTW adjustment revealed that the OAC group had significantly fewer thromboembolic events (incidence rate, 0.35 [95% CI, 0.20-0.52] vs 0.74 [95% CI, 0.42-1.13] per 100 person-years; log-rank *P* = .04) and more major bleeding events (incidence rate, 0.64 [95% CI, 0.42-0.89] vs 0.22 [95% CI, 0.05-0.44] per 100 person-years; *P* < .001) than the non-OAC group (eFigure 6A and B in Supplement 1), whereas no significant difference in all-cause death (incidence rate, 0.69 [95% CI, 0.47-0.94] vs 1.09 [95% CI, 0.67-1.56] per 100 person-years; log-rank *P* = .10) was observed between the 2 groups (eFigure 6C in Supplement 1).

An alternative IPTW analysis based on a landmark period of 6 months demonstrated no notable differences in thromboembolism, major bleeding, and mortality between the OAC continuation (n = 1404) and OAC discontinuation groups (n = 651) (eFigure 7 in Supplement 1).

Event rates and risks of adverse events among the CHA_2_DS_2_-VASc score groups are shown in eTable 7 in Supplement 1. In the IPTW analysis, OAC discontinuation had a higher risk of thromboembolism in the group with a CHA_2_DS_2_-VASc score of 4 or greater, whereas OAC discontinuation resulted in a lower risk of bleeding among the group possessing a CHA_2_DS_2_-VASc score of 3.

## Discussion

This study examined OAC discontinuation and characteristics associated with prognoses after successful CA. A previous study reported that CA for AF may reduce the risk of thromboembolic events because of the marked reduction in the AF burden postoperatively.^4^ Thus, it is plausible that patients without recurrence after successful CA may have the option of OAC discontinuation following a certain follow-up period. A 2020 post hoc analysis of the Chinese Atrial Fibrillation Registry study demonstrated both low thromboembolic and bleeding risks after OAC discontinuation 3 months after CA.^15^ Because the differences in event rates between thromboembolic events and bleeding after OAC discontinuation in that study (0.56 and 0.19 per 100 person-years) were relatively smaller than those in our study (0.86 [95% CI, 0.45-1.35] and 0.10 [95% CI, 0.02-0.19] per 100 person-years), the determination timing of 3 months after CA seems to be somewhat earlier because the risk of AF recurrence cumulatively increases as the follow-up time is prolonged, which may be associated with the development of stroke events thereafter. In this context, a 2024 study using the Japanese nationwide administrative claims database evaluated a landmark analysis with OAC discontinuation at 6 months after CA, and the investigators reported an individual increased risk of thromboembolism and bleeding events according to CHADS_2_ scores.^16^ However, the alternative IPTW analysis of the 6-month landmark point in our study showed no significant benefit of OAC discontinuation for outcomes, suggesting that our definition of the time point (12 months after CA) may also be feasible based on the limited assessment of examinations and follow-up visits in the clinical setting, which may be in line with features of clinical practice.

Population characteristics of patients undergoing CA for AF are usually heterogeneous, contributing to different risks of thrombosis and bleeding events. Thus, it may be better to stratify the risk of outcomes individually according to specific characteristics.^12,13,16^ In this study, the interactive factors of asymptomatic AF, LVEF of less than 60%, and LAD of 45 mm or greater were the reasonable background characteristics associated with the risk of thromboembolism. Specifically, asymptomatic AF is less likely to be detected in short-term Holter monitoring due to the limited duration (eg, 24-72 hours) and frequency of monitoring, leading to underrecognized AF development and subsequent stroke occurrence.^21,22^ The issue was supported by evidence in a previous report demonstrating the feasibility of continuous rhythm monitoring-guided OAC management resulting in the absence of stroke events following CA.^23^ Given that a higher proportion of postablation AF recurrences were asymptomatic, and certain recurrences may have been underestimated during routine monitoring examinations,^24^ patients without relevant symptoms of AF may be strongly recommended to continue OACs, even after successful CA, although our study assessed AF symptoms before CA only and asymptomatic recurrence was reported to increase after CA previously.^25^ With regard to LVEF (<60%) and LAD (≥45 mm), it is well known that patients with poor cardiac function are at increased risk of thromboembolism,^26^ and left atrial morphology and increased size are markedly associated with the risk of thromboembolism.^27,28^ In contrast, the HAS-BLED score itself was clinically developed to evaluate the risk of bleeding after OAC administration in patients with AF.^29,30^ Surprisingly, CHADS_2_ and CHA_2_DS_2_-VASc scores were not independently associated with the outcomes in our study.^12,16^ Moreover, no statistically significant difference was observed in patients with a previous ischemic stroke. The relatively small sample size, with few occurrences of events in each subgroup, may be a possible reason for these findings. Conversely, Kanaoka et al^16^ recently reported that the benefits and risks of continuing OAC therapy after CA differ based on the patient’s CHADS_2_ score (≥3 vs <3). A large-scale sample in their study may be a reason to stratify the different risks according to the CHADS_2_ score. Nevertheless, the option of decision-making for discontinuing OAC after a certain period of CA was feasible and important to consider the overall balance of risk and benefit, which is consistent with our study concept, regardless of discontinuation timing. Notably, compared with the OAC continuation group, the OAC discontinuation group had a higher incidence of thromboembolic events despite the lower recurrence after the landmark period. A possible explanation may be the relatively long duration of the regular visit follow-up for those patients owing to successful CA and the absence of OAC prescribing; this trend may reduce the chance of detecting asymptomatic AF recurrence that may be attributed to subsequent thromboembolic events. This finding was also supported by a previous study reporting that AF incidence was not always present at the time of the stroke event, and some AFs occurred asymptomatically before the event.^31^

In this study, the distinct results of thromboembolic events in the PS-matched analysis compared with the IPTW analysis may be due to a relatively low-risk population extracted from the matched cohort. OAC discontinuation may be safe in patients with a low risk profile, as shown in the PS-matched cohort, whereas some specific characteristics (eg, asymptomatic AF and decreased cardiac function) may prompt OAC continuation to prevent stroke events. Thus, a better balance of the risk estimation between 2 conflicting events should be constructed based on the specific characteristics beforehand.

### Limitations

This study has some limitations. As a retrospective, single-center study, it may have had an insufficient sample size owing to the low estimated event rates. Specifically, the subgroup analyses had limited statistical power due to the small number of events. There was considerable bias in the patient background characteristics between the 2 groups, and the decision to discontinue OACs was entirely dependent on the physician’s discretion. The long 15-year span of study population recruitment involved a different ablation approach and therapeutic techniques; in particular, a time-dependent bias in the approach of postprocedural OAC usage and development of guidelines may have affected the outcomes. Information on OAC administration before and after 12 months was not included in the classification of the landmark analysis, indicating some discrepancies in OAC administration in the assigned groups. Although we set a long duration of 12 months before OAC discontinuation in this study, subclinical recurrence and asymptomatic AF that could not have been documented in the limited ECG monitoring may have occurred. We did not assess the effect of adherence to OACs, suboptimal or overdose of OACs, and management of the therapeutic range of warfarin, which are possibly associated with an additional underlying risk of events. Moreover, we assessed left atrial dilation using LAD only and did not incorporate left atrial volume index, which could have provided a more precise evaluation of atrial enlargement.

## Conclusions

In this retrospective cohort study, thromboembolic risk increased after OAC discontinuation at 12 months after CA for AF, whereas the increased risk of major bleeding was relevant in the OAC continuation group. Discontinuation of OACs was determined by a balanced risk of thromboembolic and bleeding events, in line with specific characteristics. Further research on a prospective randomized study is necessary to determine the optimal use of OACs after CA for AF.

## Notes

Supplement 1.
eMethods
**eTable 1.** Details of Each Event and Comparison Between the OAC Discontinuation and Continuation Groups **eTable 2.** Incidence Rates of Adverse Events According to the Analysis Method **eTable 3.** Absolute Risk and Relative Risk Differences for Adverse Events **eTable 4.** OAC Administration and AF Recurrence Rates at the Time of the Events **eTable 5.** Baseline Characteristics of OAC Discontinuation and Continuation Groups Following PS-Matched Analysis **eTable 6.** Details of Each Event and Comparison of the OAC Discontinuation and Continuation Groups After PS-Matched Analysis **eTable 7.** Event Rates and Risks of Adverse Events in CHA_2_DS_2_-VASc Score Groups **eFigure 1.** Distribution of PSs Before (A) and After (B) IPTW Analysis to Adjust the Weighted Balance of Baseline Characteristics Between the OAC Discontinuation and Continuation Groups **eFigure 2.** SMD Between the OAC Discontinuation and Continuation Groups Before and After IPTW Analysis **eFigure 3.** Kaplan-Meier Curve Analysis of Thromboembolic Events (A), Major Bleeding Events (B), and All-Cause Death (C) Between the OAC Discontinuation and Continuation Groups Following PS-Matched Analysis **eFigure 4.** Subgroup Analysis of All-Cause Death Between the OAC Discontinuation and Continuation Groups After IPTW Analysis **eFigure 5.** Subgroup Analysis of Thromboembolic Events (A), Major Bleeding Events (B), and All-Cause Death (C) Between the OAC Discontinuation and Continuation Groups After PS-Matched Analysis **eFigure 6.** Kaplan-Meier Curve Analysis of Thromboembolic Events (A), Major Bleeding Events (B), and All-Cause Death (C) Between the OAC Discontinuation and Continuation Groups in the As-Treated Analysis **eFigure 7.** Kaplan-Meier Curve Analysis of Thromboembolic Events (A), Major Bleeding Events (B), and All-Cause Death (C) Between the OAC Discontinuation (N=651) and Continuation (N=1404) Groups After IPTW Analysis With a Landmark Period of 6 Months
eReferences

Supplement 2.
Data Sharing Statement
