# Supplementary material for: Discontinuation of Oral Anticoagulation After Successful Atrial Fibrillation Ablation
Source: JAMA Netw Open. 2025 Mar 21;8(3):e251320. doi: 10.1001/jamanetworkopen.2025.1320 (PMC11929034; doi:10.1001/jamanetworkopen.2025.1320)
Supplement: Supplement 1. — eMethods eTable 1. Details of Each Event and Comparison Between the OAC Discontinuation and Continuation Groups eTable 2. Incidence Rates of Adverse Events According to the Analysis Method eTable 3. Absolute Risk and Relative Risk Differences for Adverse Events eTable 4. OAC Administration and AF Recurrence Rates at the Time of the Events eTable 5. Baseline Characteristics of OAC Discontinuation and Continuation Groups Following PS-Matched Analysis eTable 6. Details of Each Event and Comparison of the OAC Discontinuation and Continuation Groups After PS-Matched Analysis eTable 7. Event Rates and Risks of Adverse Events in CHA2DS2-VASc Score Groups eFigure 1. Distribution of PSs Before (A) and After (B) IPTW Analysis to Adjust the Weighted Balance of Baseline Characteristics Between the OAC Discontinuation and Continuation Groups eFigure 2. SMD Between the OAC Discontinuation and Continuation Groups Before and After IPTW Analysis eFigure 3. Kaplan-Meier Curve Analysis of Thromboembolic Events (A), Major Bleeding Events (B), and All-Cause Death (C) Between the OAC Discontinuation and Continuation Groups Following PS-Matched Analysis eFigure 4. Subgroup Analysis of All-Cause Death Between the OAC Discontinuation and Continuation Groups After IPTW Analysis eFigure 5. Subgroup Analysis of Thromboembolic Events (A), Major Bleeding Events (B), and All-Cause Death (C) Between the OAC Discontinuation and Continuation Groups After PS-Matched Analysis eFigure 6. Kaplan-Meier Curve Analysis of Thromboembolic Events (A), Major Bleeding Events (B), and All-Cause Death (C) Between the OAC Discontinuation and Continuation Groups in the As-Treated Analysis eFigure 7. Kaplan-Meier Curve Analysis of Thromboembolic Events (A), Major Bleeding Events (B), and All-Cause Death (C) Between the OAC Discontinuation (N=651) and Continuation (N=1404) Groups After IPTW Analysis With a Landmark Period of 6 Months eReferences [file jamanetwopen-e251320-s001.pdf]

Supplemental Online Content

Iwawaki T, Yanagisawa S, Inden Y, et al. Discontinuation of oral anticoagulation after successful atrial fibrillation ablation. *JAMA Netw Open*. 2025;8(3):e251320. doi:10.1001/jamanetworkopen.2025.1320

eMethods

eTable 1. Details of Each Event and Comparison Between the OAC Discontinuation and Continuation Groups

eTable 2. Incidence Rates of Adverse Events According to the Analysis Method

eTable 3. Absolute Risk and Relative Risk Differences for Adverse Events

eTable 4. OAC Administration and AF Recurrence Rates at the Time of the Events

eTable 5. Baseline Characteristics of OAC Discontinuation and Continuation Groups Following PS-Matched Analysis

eTable 6. Details of Each Event and Comparison of the OAC Discontinuation and Continuation Groups After PS-Matched Analysis

eTable 7. Event Rates and Risks of Adverse Events in CHA_2DS_2-VASc Score Groups

eFigure 1. Distribution of PSs Before (A) and After (B) IPTW Analysis to Adjust the Weighted Balance of Baseline Characteristics Between the OAC Discontinuation and Continuation Groups

eFigure 2. SMD Between the OAC Discontinuation and Continuation Groups Before and After IPTW Analysis

eFigure 3. Kaplan-Meier Curve Analysis of Thromboembolic Events (A), Major Bleeding Events (B), and All-Cause Death (C) Between the OAC Discontinuation and Continuation Groups Following PS-Matched Analysis

eFigure 4. Subgroup Analysis of All-Cause Death Between the OAC Discontinuation and Continuation Groups After IPTW Analysis

eFigure 5. Subgroup Analysis of Thromboembolic Events (A), Major Bleeding Events (B), and All-Cause Death (C) Between the OAC Discontinuation and Continuation Groups After PS-Matched Analysis

eFigure 6. Kaplan-Meier Curve Analysis of Thromboembolic Events (A), Major Bleeding Events (B), and All-Cause Death (C) Between the OAC Discontinuation and Continuation Groups in the As-Treated Analysis

© 2025 Iwawaki T et al. *JAMA Network Open*.

eFigure 7. Kaplan-Meier Curve Analysis of Thromboembolic Events (A), Major Bleeding Events (B), and All-Cause Death (C) Between the OAC Discontinuation (N = 651) and Continuation (N = 1404) Groups After IPTW Analysis With a Landmark Period of 6 Months

eReferences

This supplemental material has been provided by the authors to give readers additional information about their work.

© 2025 Iwawaki T et al. *JAMA Network Open*.

eMethods

Examinations and ablation approach

All patients were admitted the day before the procedure and were examined using blood sampling, 12-lead electrocardiography (ECG), and transthoracic and transesophageal echocardiography. The patient characteristics including blood tests, echocardiography, ECG, and medication status were measured on the day before catheter ablation. The $CHADS_2$ and CHA_2DS_2-VASc scores and HAS-BLED scores were calculated as previously described.[1-3] The HAS-BLED score did not include prothrombin time-international normalized ratio (INR) when the patients took direct oral anticoagulants (OAC); however, for patients taking warfarin, significantly unstable INR values (labile INR) were considered as part of the score. Enhanced computed tomography was performed to evaluate the left atrial (LA) morphology and exclude the possibility of thrombus formation. Anti-arrhythmic drugs (AAD) were discontinued for five half-lives before ablation, except for amiodarone. All patients continued OAC uninterruptedly during the catheter ablation (CA) procedure.[4]

The ablation system utilized a 3-dimensional mapping system, CARTO (Johnson and Johnson Medical, Biosense Webster, Inc., Irvine, CA, USA), and EnSite System (St. Jude Medical, Saint Paul, MN, USA). Radiofrequency, cryoballoon, hot balloon, and laser balloon ablations were performed during the study period. The ablation energy and type of application were chosen by the attending physician based on atrial fibrillation (AF) type, LA morphology, and patient backgrounds, with most cases being radiofrequency followed by cryoballoon ablation.[5] For patients with paroxysmal AF or early persistent AF, only pulmonary vein isolation was performed. However, in patients with long-standing persistent AF or atrial flutter, linear and substrate ablations were applied. If the AF rhythm persisted at the end of CA, external cardioversion was performed to restore the sinus rhythm.

Follow-up examinations to detect recurrence

All patients underwent continuous ECG monitoring for 3 days after the procedure. The nursing staff instructed all patients to check their pulse to identify an abnormality of the rhythm promptly during the hospitalization. After discharge, patients were typically scheduled to visit our institution's outpatient clinic specialized in arrhythmia and electrophysiology at 1, 3, 6, 9, and 12 months after CA and general practitioner at a minimum every month. At each visit, all patients were examined using a 3-min surface 12-lead ECG. All patients underwent 24-h Holter ECG monitoring after one month and more. When AF recurrence was suspected based on the patient's given consultation, additional Holter monitoring and repeated ECG testing within a short duration of follow-up were performed to detect subclinical recurrences. Patients were advised to promptly visit the hospital if they experienced any concerning symptoms. Additionally, we monitored all medical records for AF recurrence, for instance, when patients visited the emergency room or

© 2025 Iwawaki T et al. *JAMA Network Open*.

non-cardiology outpatient clinics. All AF recurrences detected in the examination testing and recurrence date have been reviewed by at least two independent investigators. The first recurrence day following a blanking period of CA was set to an index date for the statistical, survival analysis. After the landmark period, patients continued to receive regular ECG monitoring at our institution's outpatient clinic and general practitioner. AADs were not typically resumed unless patients had a recurrence during the blanking period.

Definitions of outcomes

Thromboembolic and major bleeding events, all-cause deaths, and AF recurrence occurring over 12 months after CA were defined as the primary outcomes. Major bleeding was defined according to the International Society of Thrombosis and Hemostasis definition of bleeding complications[6] which includes fatal bleeding, symptomatic bleeding in a critical area or organ needing urgent hospitalizations, and severe bleeding loss requiring transfusions. Thromboembolism included ischemic stroke, systemic embolism, and transient ischemic attacks confirmed by clinical and imaging evaluation. The asymptomatic lacunar lesion accidentally detected in a brain imaging study was not counted for the events. All-cause death was evaluated from medical records and telephone interviews, if applicable, and primary pathology, which may have been mostly associated with the corresponding death, was determined as the major cause of death. AF recurrence was defined as the first AF/atrial tachycardia lasting >30 sec on examination testing after the blanking period of CA. These outcomes were retrospectively identified using comprehensive reviews of medical records, imaging studies, and physician documentation. The primary endpoint was defined as thromboembolic events in the main analysis and as-treated analysis.

Statistical analysis

For an inverse probability of treatment weighting (IPTW) analysis to weigh each individual propensity score (PS) via the inverse probability of receiving either treatment, PS was calculated using a logistic regression model with OAC discontinuation as the dependent variable, which included the following covariates: age, sex, body mass index, AF symptoms, type of AF, AADs use, type of OACs, anti-platelet drug use, ablation methods, $CHADS_2$ score, CHA_2DS_2-VASc score, HAS-BLED score, left ventricular ejection fraction, left atrial diameter, history of heart failure and ischemic stroke, and history of device implantation. All these parameters had been selected to be potentially associated with OAC discontinuation in the field of general clinical medicine before the entire analysis. Based on the balance and limited sample size of the model, we could not include all parameters with significant differences presented in Table 1 in the PS model. Since the $CHADS_2$ scores, CHA_2DS_2-VASc scores, and HAS-BLED scores change mostly parallelly with a strong correlation, the analysis model might have a risk of collapsing with

© 2025 Iwawaki T et al. *JAMA Network Open*.

multicollinearity when we use these continuous variables in the same model. Thus, we have alternatively defined categorical values of $CHADS_2$ score ≥2, CHA_2DS_2-VASc scores ≥3, and HAS-BLED score ≥2 for the PS matching model which could be sufficient to develop the analysis model. IPTW used the PS to balance baseline patient characteristics in the OAC continuation and discontinuation groups by weighting each individual by the inverse probability of receiving his/her actual treatment. Weights were calculated for each individual as 1/PS for the exposed group and 1/(1−PS) for the unexposed group. The balance between the groups before and after matching was assessed using standardized mean differences, and those <0.10 were considered a negligible imbalance. After the calculation of the weights, the weights can be incorporated into a weighted Cox regression model for a time-to-event outcome to obtain estimates adjusted for confounders. Kaplan–Meier survival curves were generated to visualize the survival rates for outcomes, and the log-rank test was performed to evaluate the statistical significance of the difference between the weighted cumulative incidence curves of the two groups with IPTW analysis. A landmark analysis with a period of 12 months was conducted for any comparison to reduce immortal time bias.

In the PS-matched analysis, 1:1 nearest-neighbor greedy matching was performed based on the calculated score individually, and we used caliper widths with 0.2 of a standard deviation of the logit of PS. The weighted balance of the PS according to the two populations based on the as-treated approach was adjusted again using IPTW analysis, and the prognosis was compared using a weighted cumulative incidence rate. All analyses were performed using the SPSS version 29.0 (SPSS Inc., Chicago, IL, USA) and R software (version 4.3.3).

© 2025 Iwawaki T et al. *JAMA Network Open*.

eTable 1. Details of Each Event and Comparison Between the OAC Discontinuation and Continuation Groups

Parameters	*OAC discontinuation (n=899)*	*OAC continuation (n=922)*	*P-value*
Thromboembolic events, No. (%)	20 (2.2)	23 (2.5)	0.71
Cerebral infarction	14 (1.5)	20 (2.2)	0.34
TIA	6 (0.7)	3 (0.3)	0.30
Major bleeding events, No. (%)	5 (0.5)	36 (3.9)	<.001
Cerebral hemorrhage	2 (0.2)	12 (1.3)	0.008
Gastrointestinal hemorrhage	3 (0.3)	23 (2.5)	<.001
Spinal hemorrhage	0 (0)	1 (0.1)	0.32
All-cause death, No. (%)	21 (2.3)	50 (5.4)	0.001
Cardiac disease	0 (0)	12 (1.3)	0.001
Infection	4 (0.4)	6 (0.7)	0.55
Bleeding disease	0 (0)	6 (0.7)	0.01
Malignant tumor	9 (1.0)	13 (1.4)	0.43
Others	4 (0.4)	3 (0.3)	0.68
Unknown	4 (0.4)	10 (1.1)	0.12
AF recurrence, No. (%)	155 (17.2)	194 (21.0)	0.04

AF, atrial fibrillation; OAC, oral anticoagulant; TIA, transient ischemic attack.

© 2025 Iwawaki T et al. *JAMA Network Open*.

eTable 2. Incidence Rates of Adverse Events According to the Analysis Method

Parameters	Incidence rate/100 person-years		
	OAC (-)	***OAC (+)***	***P-value (log-rank test)***
**Thromboembolic events**			
Crude population	0.49 (0.28-0.73)	0.49 (0.29-0.68)	0.86
IPTW analysis	0.86 (0.45-1.35)	0.37 (0.22-0.54)	0.04
PS-matched analysis	0.61 (0.32-0.91)	0.39 (0.16-0.62)	0.24
As-treated analysis	0.74 (0.42-1.13)	0.35 (0.20-0.52)	0.04
IPTW analysis (6 months)	0.89 (0.36-1.57)	0.43 (0.29-0.59)	0.15
**Major bleeding events**			
Crude population	0.12 (0.02-0.23)	0.76 (0.52-1.01)	<.001
IPTW analysis	0.10 (0.02-0.19)	0.65 (0.43-0.90)	<.001
PS-matched analysis	0.19 (0.02-0.35)	0.61 (0.33-0.90)	0.02
As-treated analysis	0.22 (0.05-0.44)	0.64 (0.42-0.89)	<.001
IPTW analysis (6 months)	0.24 (0.03-0.53)	0.54 (0.37-0.73)	0.06
**All-cause death**			
Crude population	0.43 (0.22-0.65)	0.96 (0.70-1.22)	0.006
IPTW analysis	0.82 (0.58-1.09)	0.99 (0.40-1.88)	0.67
PS-matched analysis	0.62 (0.29-0.94)	0.97 (0.63-1.31)	0.30
As-treated analysis	1.09 (0.67-1.56)	0.69 (0.47-0.94)	0.10
IPTW analysis (6 months)	0.60 (0.16-1.29)	0.81 (0.62-1.02)	0.53

Values are expressed as 95% confidence intervals. IPTW, inverse probability treatment weighting; OAC, oral anticoagulant; PS, propensity score.

© 2025 Iwawaki T et al. *JAMA Network Open*.

eTable 3. Absolute Risk and Relative Risk Differences for Adverse Events

Parameters	IPTW analysis		PS-matched analysis		As-treated analysis	
**Thromboembolic events**		P-value		P-value		P-value
Absolute risk difference (95%CI)	0.49 (0.01 to 0.98)	0.05	0.19 (-0.19 to 0.42)	0.44	0.39 (0.01 to 0.77)	0.05
Relative risk difference (95%CI)	2.43 (1.23 to 4.79)	0.01	1.58 (0.73 to 3.40)	0.25	2.21 (1.15 to 4.26)	0.02
**Major bleeding events**						
Absolute risk difference (95%CI)	-0.55 (-0.81 to -0.30)	<.001	-0.46 (-0.79 to -0.10)	0.02	-0.42 (-0.72 to -0.11)	<.001
Relative risk difference (95%CI)	0.15 (0.06 to 0.39)	<.001	0.31 (0.11 to -0.84)	0.02	0.35 (0.13 to 0.92)	0.03
**All-cause death**						
Absolute risk difference (95%CI)	0.18 (-0.63 to 0.99)	0.67	-0.31 (-0.79 to 0.19)	0.28	0.40 (-0.10 to 0.89)	0.12
Relative risk difference (95%CI)	1.22 (0.53 to 2.82)	0.65	0.73 (0.40 to 1.33)	0.30	1.63 (0.96 to 2.77)	0.07

Values are expressed as incidence rate/100 person-years. OAC continuation was used as a reference.

CI, confidence interval; IPTW, inverse probability of treatment weighting; OAC, oral anticoagulant; PS, propensity score.

© 2025 Iwawaki T et al. *JAMA Network Open*.

eTable 4. OAC Administration and AF Recurrence Rates at the Time of the Events

**OAC administration rates at the time of events, No. (%)**	***OAC discontinuation group***	***OAC continuation group***	***P-value***
Thromboembolic events	4/20 (20.0)	18/23 (78.3)	<.001
Major bleeding events	2/5 (40.0)	34/36 (94.4)	0.09
All-cause death	3/21 (14.3)	40/50 (80.0)	<.001
**AF recurrence rates, No. (%)**			
Thromboembolic events	4/20 (20.0)	7/23 (30.0)	0.45
Major bleeding events	3/5 (60.0)	8/36 (22.0)	0.08
All-cause death	3/21 (14.3)	4/50 (8.0)	0.43

Of 349 patients with recurrent AF, 172 (49.3%) were not taking OACs at the time of recurrence. Of these, 128 patients (74.4%) resumed OACs after that.

AF, atrial fibrillation; OAC, oral anticoagulant.

© 2025 Iwawaki T et al. *JAMA Network Open*.

eTable 5. Baseline Characteristics of OAC Discontinuation and Continuation Groups Following PS-Matched Analysis

Characteristics	*OAC discontinuation (n=550)*	*OAC continuation (n=550)*	*P-value*	*SMD (95%CI)*
Age, mean (SD), y	64.3 (10.3)	64.2 (10.3)	0.91	0.01 (-0.11 to 0.12)
Sex, No. (%)				
Male	408 (74.2)	405 (73.6)	0.84	0.01 (-0.05 to 0.07)
Female	142 (25.8)	145 (26.4)	0.84	0.01 (-0.05 to 0.07)
BMI, mean (SD)	24.0 (3.6)	24.1 (3.8)	0.64	0.03 (-0.09 to 0.14)
AF duration, mean (SD), y	2.6 (3.7)	3.1 (4.4)	0.05	0.12 (0.00 to 0.24)
AF type, No. (%)				
Paroxysmal	363 (66.0)	355 (64.5)	0.61	0.03 (-0.03 to 0.09)
Non-paroxysmal	187 (34.0)	195 (35.5)	0.61	0.03 (-0.03 to 0.09)
Symptomatic	379 (68.9)	371 (67.5)	0.61	0.03 (-0.03 to 0.09)
Hemodialysis, No. (%)	3 (0.5)	4 (0.7)	0.71	0.02 (-0.04 to 0.08)
Thrombosis score, mean (SD)				
$CHADS_2$ score	1.01 (0.87)	0.99 (0.85)	0.57	0.02 (-0.09 to 0.14)
CHA_2DS_2-VASc score	1.91 (1.35)	1.86 (1.25)	0.53	0.04 (-0.08 to 0.16)
Bleeding score (HAS-BLED), mean (SD)	1.34 (0.99)	1.35 (0.92)	0.85	0.01 (-0.11 to 0.12)
Laboratory data				
BNP level, median (IQR), pg/mL	51.6 (24.2–116.8)	59.4 (26.1–115.1)	0.21	0.07 (0.00 to 0.23)
eGFR, mean (SD), mL/min/1.73 m^2	70.2 (23.2)	67.9 (16.4)	0.06	0.11 (0.00 to 0.23)
PT-INR, mean (SD)	1.48 (0.60)	1.50 (0.53)	0.65	0.04 (-0.08 to 0.15)
APTT, mean (SD), s	41.2 (10.6)	41.3 (9.5)	0.80	0.01 (-0.11 to 0.13)
D-dimer, mean (SD), µg/mL	0.73 (2.15)	0.63 (0.69)	0.30	0.06 (-0.06 to 0.18)
Echocardiographic data				
LVEF, mean (SD), %	61.8 (8.9)	61.2 (9.2)	0.33	0.07 (-0.05 to 0.18)
LAD, mean (SD), mm	39.3 (6.0)	39.3 (6.7)	0.90	<.001 (-0.12 to 0.12)
MR (moderate or greater) n (%)	11 (2.0)	16 (2.9)	0.33	0.06 (0.00 to 0.11)
TR (moderate or greater) n (%)	13 (2.4)	15 (2.7)	0.71	0.02 (-0.03 to 0.08)
Comorbidity, No. (%)				
Hypertension	293 (53.3)	290 (52.7)	0.86	0.01 (-0.05 to 0.07)
Diabetes	77 (14.0)	71 (12.9)	0.60	0.03 (-0.03 to 0.09)
Heart failure	62 (11.3)	65 (11.8)	0.78	0.02 (-0.04 to 0.08)
Ischemic heart disease	44 (8.0)	32 (5.8)	0.15	0.09 (0.03 to 0.15)
Ischemic stroke	22 (4.0)	17 (3.1)	0.42	0.05 (-0.01 to 0.11)
Vascular disease	49 (8.9)	32 (5.8)	0.05	0.12 (0.06 to 0.18)

© 2025 Iwawaki T et al. *JAMA Network Open*.

Characteristics	*OAC discontinuation (n=550)*	*OAC continuation (n=550)*	*P-value*	*SMD (95%CI)*
Device implantation, No. (%)				
Pacemaker	3 (0.5)	8 (1.5)	0.13	0.09 (0.03 to 0.15)
ICD	0 (0)	9 (1.6)	0.003	0.18 (0.19 to 0.31)
CRT	1 (0.2)	5 (0.9)	0.10	0.10 (0.04 to 0.16)
Ablation procedure, No. (%)				
PV isolation	550 (100)	550 (100)	>.99	N/A
CTI ablation	389 (70.7)	391 (71.1)	0.89	<.001 (-0.05 to 0.07)
Bottom line ablation	116 (21.1)	125 (22.7)	0.51	0.04 (-0.02 to 0.10)
Roof line ablation	155 (28.2)	168 (30.5)	0.39	0.05 (-0.01 to 0.11)
SVC isolation	25 (4.5)	30 (5.5)	0.49	0.04 (-0.02 to 0.10)
MI ablation	81 (14.7)	100 (18.2)	0.12	0.09 (0.03 to 0.15)
Radiofrequency ablation	434 (78.9)	449 (81.6)	0.26	0.07 (0.00 to 0.13)
Cryoballoon ablation	100 (18.2)	79 (14.4)	0.09	0.10 (0.04 to 0.16)
Hot balloon ablation	13 (2.4)	17 (3.1)	0.46	0.04 (-0.01 to 0.10)
Laser balloon ablation	3 (0.5)	5 (0.9)	0.48	0.04 (-0.01 to 0.10)
Medication, No. (%)				
Warfarin	203 (36.9)	202 (36.7)	0.95	<.001 (-0.06 to 0.06)
DOAC	347 (63.1)	348 (63.3)	0.95	<.001 (-0.06 to 0.06)
Antiplatelet drug	57 (10.4)	49 (8.9)	0.41	0.05 (-0.01 to 0.11)
ACE-I or ARB	201 (36.5)	217 (39.5)	0.32	0.06 (0.00 to 0.12)
β-Blocker	228 (41.5)	251 (45.6)	0.16	0.08 (0.03 to 0.14)
Loop diuretic	53 (9.6)	55 (10.0)	0.84	0.01 (-0.05 to 0.07)
MRA	24 (4.4)	44 (8.0)	0.01	0.15 (0.01 to 0.21)
AAD (I)	189 (34.4)	186 (33.8)	0.85	0.01 (-0.05 to 0.07)
AAD (III)	49 (8.9)	75 (13.6)	0.01	0.15 (0.09 to 0.21)

AAD, antiarrhythmic drug; ACEI, angiotensin-converting enzyme inhibitor; AF, atrial fibrillation; APTT, activated partial thromboplastin time; ARB, angiotensin II receptor blocker; BMI, body mass index (calculated as weight in kilograms divided by height in meters squared); BNP, brain natriuretic peptide; $CHADS_2$, congestive heart failure, hypertension, age, diabetes, and stroke; CHA_2DS_2-VASc, congestive heart failure, hypertension, aged 75 years or older, diabetes, stroke–vascular disease, aged 65 to 74 years, and female sex; CI, confidence interval; CRT, cardiac resynchronization therapy; CTI, cavo tricuspid isthmus; DOAC, direct oral anticoagulant; eGFR, estimated glomerular filtration rate; HAS-BLED, hypertension, kidney or liver disease, stroke history, prior bleeding, unstable international normalized ratio, aged older than 65 years, and drug or alcohol use; ICD, implantable cardioverter defibrillator; LAD, left atrial diameter; LVEF, left ventricular ejection fraction; MI, mitral isthmus; MR, mitral valve regurgitation; MRA, mineralocorticoid receptor antagonist; NA, not applicable; OAC, oral anticoagulant; PT-INR, prothrombin time-international normalized ratio; PV,

© 2025 Iwawaki T et al. *JAMA Network Open*.

pulmonary vein; SMD, standardized mean difference; SVC, superior vena cava; TR, tricuspid valve regurgitation.

© 2025 Iwawaki T et al. *JAMA Network Open*.

eTable 6. Details of Each Event and Comparison of the OAC Discontinuation and Continuation Groups After PS-Matched Analysis

**Parameters**	***OAC discontinuation (n=550)***	***OAC continuation (n=550)***	***P-value***
Thromboembolic events, No. (%)	16 (2.9)	11 (2.0)	0.33
Cerebral infarction	11 (2.0)	9 (1.6)	0.65
TIA	5 (0.9)	2 (0.4)	0.26
Major bleeding events, No. (%)	5 (0.9)	17 (3.1)	0.01
Cerebral hemorrhage	2 (0.4)	7 (1.3)	0.09
Gastrointestinal hemorrhage	3 (0.5)	9 (1.6)	0.08
Spinal hemorrhage	0 (0)	1 (0.2)	0.32
All-cause death, No. (%)	18 (3.2)	26 (4.7)	0.22
Cardiac disease	0 (0)	6 (1.1)	0.01
Infection	4 (0.7)	6 (1.1)	0.53
Bleeding disease	0 (0)	0 (0)	N/A
Malignant tumor	8 (1.5)	8 (1.5)	>.99
Others	2 (0.4)	1 (0.2)	0.56
Unknown	4 (0.7)	5 (0.9)	0.74
AF recurrence, No. (%)	100 (18.2)	123 (22.4)	0.09

AF, atrial fibrillation; OAC, oral anticoagulant; PS, propensity score; TIA, transient ischemic attack.

© 2025 Iwawaki T et al. *JAMA Network Open*.

eTable 7. Event Rates and Risks of Adverse Events in CHA_2DS_2-VASc Score Groups

Parameters	IPTW analysis				PS-matched analysis				As-treated analysis			
	OAC (-)	OAC (+)	HR (95%CI)	P-value	OAC (-)	OAC (+)	HR (95%CI)	P-value	OAC (-)	OAC (+)	HR (95%CI)	P-value
**Thromboembolic events**												
CHA_2DS_2-VASc= 0	0 /267 (0)	1/75 (1.3)	N/A	N/A	0/85 (0)	1/75 (1.3)	N/A	N/A	0/268 (0)	1/74 (1.4)	N/A	N/A
CHA_2DS_2-VASc= 1	5/263 (1.9)	2/182 (1.1)	2.696 (0.588-12.363)	0.20	3/157 (1.9)	1/152 (0.7)	2.636 (0.268-25.901)	0.41	5/274 (1.8)	2/171 (1.1)	3.132 (0.652-15.06)	0.15
CHA_2DS_2-VASc= 2	2/174 (1.1)	3/222 (1.4)	0.501 (0.086-2.909)	0.44	1/124 (0.8)	3/171 (1.8)	0.413 (0.043-3.975)	0.44	2/172 (1.2)	3/224 (1.3)	0.552 (0.098-2.776)	0.45
CHA_2DS_2-VASc= 3	7/120 (5.8)	7/189 (3.7)	2.836 (0.928-8.662)	0.07	7/111 (6.3)	4/95 (4.2)	1.403 (0.409-4.816))	0.59	8/122 (6.6)	6/187 (6.6)	3.264 (1.113-9.572)	0.03
CHA_2DS_2-VASc≥ 4	6/75 (8.0)	10/254 (3.9)	2.895 (1.027-8.165)	0.04	5/73 (6.8)	2/57 (3.5)	1.791 (0.345-9.296)	0.49	6/80 (7.5)	10/249 (4.0)	1.965 (0.691-5.584)	0.21
**Major bleeding events**												
CHA_2DS_2-VASc= 0	1/267 (0.3)	1/75 (1.3)	1.677 (0.109-25.834)	0.71	1/85 (1.2)	1/75 (1.3)	1.458 (0.091-23.318)	0.79	0/268 (0)	2/74 (2.7)	N/A	N/A
CHA_2DS_2-VASc= 1	1/263 (0.4)	4/182 (2.2)	0.130 (0.015-1.171)	0.07	1/157 (0.6)	4/152 (2.6)	0.261 (0.029-2.340)	0.23	0/274 (0)	5/171 (2.9)	N/A	N/A
CHA_2DS_2-VASc= 2	2/174 (1.1)	4/222 (1.8)	0.204 (0.032-1.295)	0.09	2/124 (1.6)	4/171 (2.3)	0.507 (0.092-2.808)	0.44	2/172 (1.2)	4/224 (1.8)	0.177 (0.017-1.85)	0.15
CHA_2DS_2-VASc= 3	1/120 (0.8)	13/189 (6.9)	0.096 (0.012-0.774)	0.03	1/111 (0.9)	5/95 (5.3)	0.155 (0.018-1.334)	0.09	1/122 (0.8)	13/187 (7.0)	0.112 (0.014-0.89)	0.04
CHA_2DS_2-VASc≥ 4	0/75 (0)	14/254 (5.5)	N/A	N/A	0/73 (0)	3/57 (5.3)	N/A	N/A	3/80 (3.8)	11/249 (4.4)	0.98 (0.272-3.536)	0.98
**All-cause death**												
CHA_2DS_2-VASc= 0	1/267 (0.4)	1/75 (1.3)	0.921 (0.084-10.127)	0.95	0/85 (0)	1/75 (1.3)	N/A	N/A	2/268 (0.7)	0/74 (0)	N/A	N/A

© 2025 Iwawaki T et al. *JAMA Network Open*.

Parameters	IPTW analysis				PS-matched analysis				As-treated analysis			
	OAC (-)	OAC (+)	HR (95%CI)	P-value	OAC (-)	OAC (+)	HR (95%CI)	P-value	OAC (-)	OAC (+)	HR (95%CI)	P-value
CHA_2DS_2-VASc= 1	5/263 (1.9)	7 /182 (3.8)	0.513 (0.152-1.730)	0.28	4/157 (2.5)	7/152 (4.6)	0.568 (0.165-1.949)	0.37	6/174 (2.2)	6/171 (3.5)	0.763 (0.233-2.499)	0.66
CHA_2DS_2-VASc= 2	7/174 (4.0)	11/222 (5.0)	2.103 (0.578-7.655)	0.26	6/124 (4.8)	9/171 (5.3)	0.768 (0.273-2.161)	0.62	6/172 (5.4)	12/224 (5.4)	0.744 (0.242-2.289)	0.61
CHA_2DS_2-VASc= 3	2/120 (14.3)	12/189 (6.3)	0.310 (0.064-1.513)	0.15	2/111 (1.8)	5/95 (5.3)	0.310 (0.060-1.600)	0.16	3/122 (2.5)	11/187 (5.9)	0.635 (0.172-2.339)	0.50
CHA_2DS_2-VASc≥ 4	6/75 (8.0)	19/254 (7.5)	0.988 (0.389-2.507)	0.98	6/73 (8.2)	4/57 (7.0)	0.907 (0.255-3.222)	0.88	12/80 (15.0)	13/249 (5.2)	3.26 (1.463-7.261)	<.001

OAC continuation was used as a reference.
CHA_2DS_2-VASc, congestive heart failure, hypertension, aged 75 years or older, diabetes, stroke–vascular disease, aged 65 to 74 years, and female sex;
CI, confidence interval; HR, hazard ratio; IPTW, inverse probability of treatment weighting; OAC, oral anticoagulant; PS, propensity score.

© 2025 Iwawaki T et al. *JAMA Network Open*.

eFigure 1. Distribution of PSs Before (A) and After (B) IPTW Analysis to Adjust the Weighted Balance of Baseline Characteristics Between the OAC Discontinuation and Continuation Groups

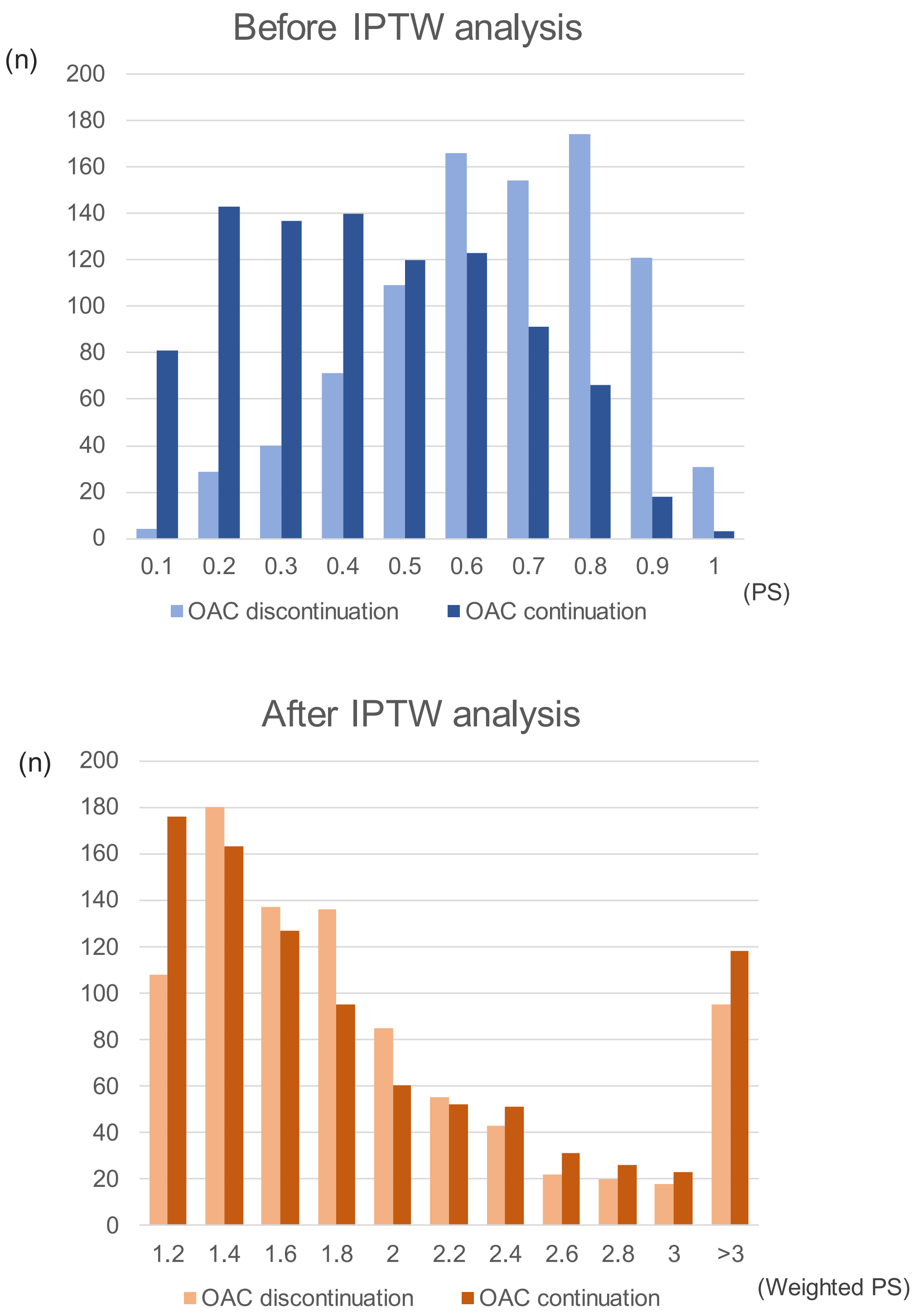

IPTW, inverse probability of treatment weighting; OAC, oral anticoagulant; PS, propensity score.

© 2025 Iwawaki T et al. *JAMA Network Open*.

eFigure 2. SMD Between the OAC Discontinuation and Continuation Groups Before and After IPTW Analysis

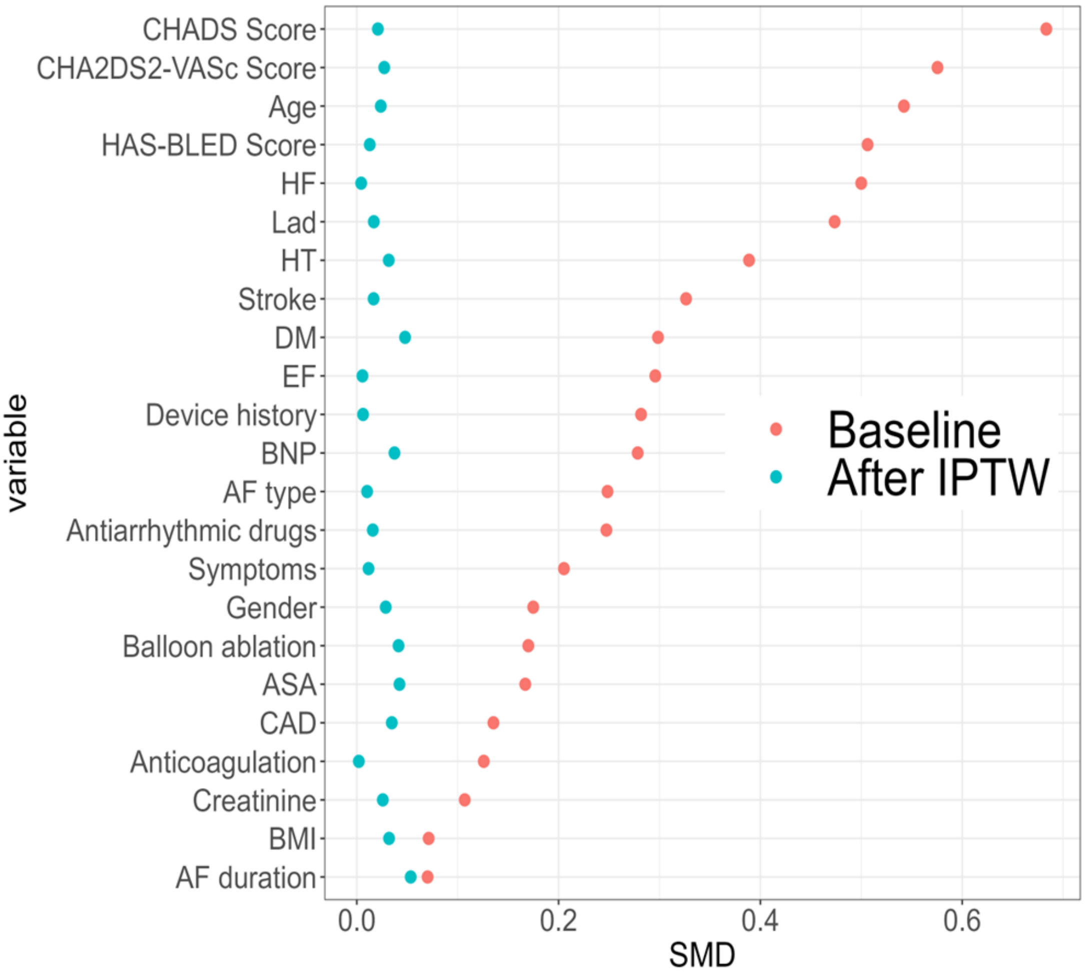

AF, atrial fibrillation; ASA, acetylsalicylic acid; BMI, body mass index; BNP, brain natriuretic peptide; CAD, coronary artery disease; $CHADS_2$, congestive heart failure, hypertension, age, diabetes, and stroke; CHA_2DS_2-VASc, congestive heart failure, hypertension, aged 75 years or older, diabetes, stroke–vascular disease, aged 65 to 74 years, and female sex; DM, diabetes; EF, ejection fraction; HF, heart failure; HT, hypertension; IPTW, inverse probability of treatment weighting; LAD, left atrial diameter; OAC, oral anticoagulant; SMD, standardized mean difference.

© 2025 Iwawaki T et al. *JAMA Network Open*.

eFigure 3. Kaplan-Meier Curve Analysis of Thromboembolic Events (A), Major Bleeding Events (B), and All-Cause Death (C) Between the OAC Discontinuation and Continuation Groups Following PS-Matched Analysis

A. Thromboembolic events

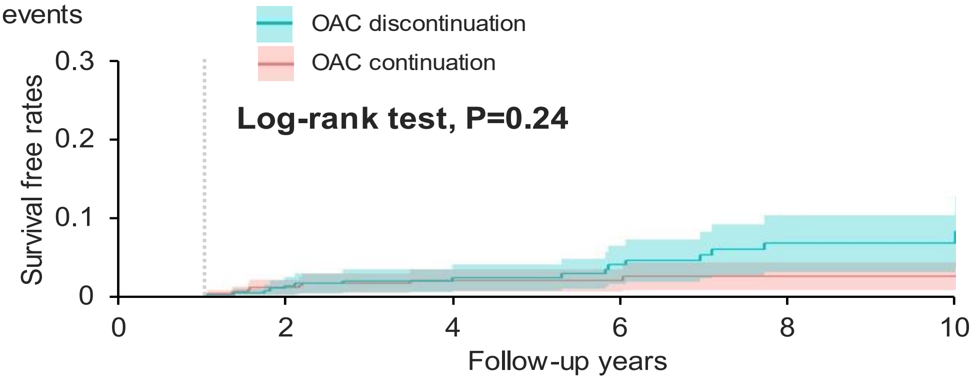

No. at risk

OAC discontinuation	550	336	249	181	126	75
OAC continuation	550	374	274	193	126	87

B. Major bleeding events

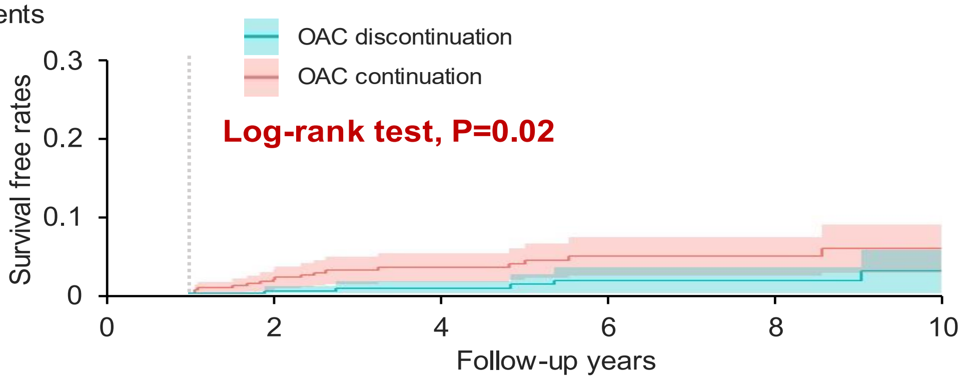

No. at risk

OAC discontinuation	550	336	249	181	126	75
OAC continuation	550	374	274	193	136	87

C. All-cause death

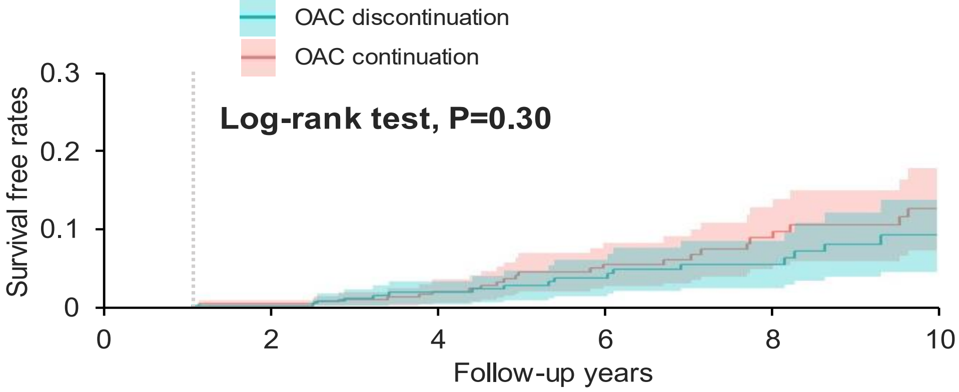

No. at risk

OAC discontinuation	550	339	253	184	132	79
OAC continuation	550	377	278	197	128	89

OAC, oral anticoagulant; PS, propensity score.

© 2025 Iwawaki T et al. *JAMA Network Open*.

eFigure 4. Subgroup Analysis of All-Cause Death Between the OAC Discontinuation and Continuation Groups After IPTW Analysis

All-cause death after IPTW analysis

Variable		*P-value*	*P for interaction*	Hazard Ratio(95%CI)
Age	≥ 65	0.25	0.12	0.680(0.354 -1.308)
	< 65	0.20		2.925(0.577 -14.840)
Sex	male	0.61	0.68	1.310(0.465 -3.689)
	female	0.99		0.995(0.356 -2.784)
Body mass index	≥ 25	0.47	0.28	0.703(0.272 -1.816)
	< 25	0.44		1.474 (0.552 -3.938)
Symptomatic AF	Yes	0.10	<.001	0.521(0.240 -1.134)
	No	0.03		3.267(1.130 -9.443)
Paroxysmal AF	Yes	0.51	0.40	1.407(0.514 -3.852)
	No	0.61		0.770(0.282 -2.097)
$CHADS_2$score	≥ 2	0.29	0.08	1.672(0.650 -4.302)
	< 2	0.11		0.550(0.263 -1.149)
CHA_2DS_2-VASc score	≥ 3	0.47	0.28	0.739(0.323 -1.687)
	< 3	0.41		1.712(0.483 -6.064)
HAS-BLED score	≥ 2	0.82	0.81	1.117(0.434 -2.874)
	< 2	0.90		0.940(0.359 -2.457)
LVEF	≥ 60	0.80	0.46	0.906(0.420 -1.955)
	< 60	0.46		1.593(0.464 -5.473)
LAD	≥ 45	0.31	0.16	0.426(0.082 -2.221)
	< 45	0.33		1.597(0.629 -4.056)
History of ischemic stroke	Yes	0.68	0.84	1.525(0.202 -11.540)
	No	0.69		1.200(0.495 -2.910)
History of HF	Yes	0.76	0.85	1.197(0.387 -3.706)
	No	0.92		1.035(0.521 -2.057)
Anti-platelet drug	Yes	N/A	N/A	none
	No	0.26		1.641(0.693 -3.883)
OAC	WF	0.68	0.39	0.854(0.400 -1.823)
	DOAC	0.45		1.632(0.463 -5.746)
History of device implantation	Yes	0.07	0.02	2.756(0.918 -8.270)
	No	0.51		0.812(0.441 -1.497)
Overall		0.65		1.218(0.526 -2.822)

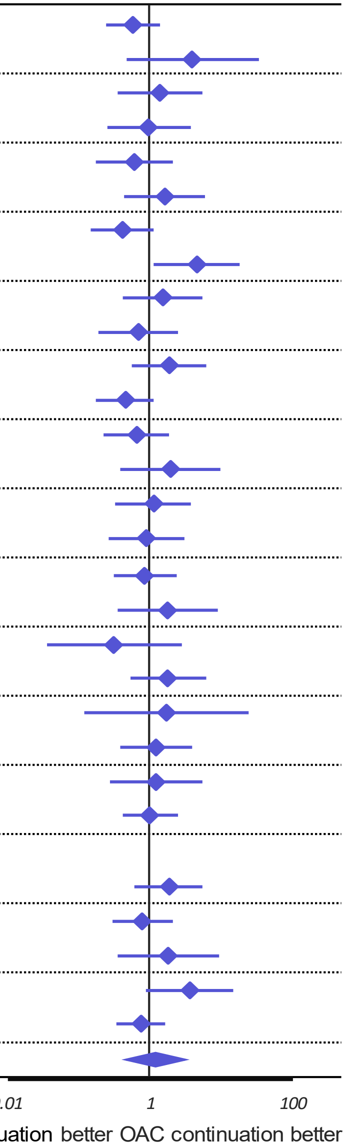

© 2025 Iwawaki T et al. *JAMA Network Open*.

OAC continuation was used as a reference.

AF, atrial fibrillation; CI, confidence interval; DOAC, direct oral anticoagulant; HF, heart failure; IPTW, inverse probability of treatment weighting; LAD, left atrial diameter; LVEF, left ventricular ejection fraction; OAC, oral anticoagulant; WF, warfarin.

© 2025 Iwawaki T et al. *JAMA Network Open*.

eFigure 5. Subgroup Analysis of Thromboembolic Events (A), Major Bleeding Events (B), and All-Cause Death (C) Between the OAC Discontinuation and Continuation Groups After PS-Matched Analysis

A. Thromboembolic events after PS-matched analysis

Variable		*P*-value	*P* for interaction	Hazard Ratio(95%CI)
Age	≥ 65	0.26	0.55	1.689(0.673 -4.241)
	< 65	>.99		1.000(0.244 -4.469)
Sex	male	0.28	0.86	1.561(0.693 -3.515)
	female	0.63		1.819(0.165 -20.096)
Body mass index	≥ 25	0.56	0.95	1.456(0.410 -5.167)
	< 25	0.33		1.615(0.614 -4.243)
Symptomatic AF	Yes	0.65	0.03	0.646(0.211 -1.974)
	No	0.02		4.366(1.217 -15.662)
Paroxysmal AF	Yes	0.38	0.96	1.544(0.587 -4.057)
	No	0.46		1.606(0.453 -5.697)
$CHADS_2$score	≥ 2	0.43	0.98	1.505(0.546 -4.149)
	< 2	0.59		1.285(0.423 -4.541)
CHA_2DS_2-VASc score	≥ 3	0.39	0.59	1.539(0.576 -4.113)
	< 3	0.94		0.950(0.255 -3.539)
HAS-BLED score	≥ 2	0.89	0.18	1.064(0.432 -2.622)
	< 2	0.11		3.648(0.736 -18.079)
LVEF	≥ 60	0.42	0.02	0.632 (0.207 -1.934)
	< 60	0.02		4.429(1.234 -15.898)
LAD	≥ 45	0.03	0.02	5.696(1.244 -26.067)
	< 45	0.49		0.694(0.247 -1.951)
History of ischemic stroke	Yes	0.85	0.63	0.768(0.048 -12.275)
	No	0.23		1.640(0.737 -3.652)
History of HF	Yes	0.20	0.38	2.852(0.570 -14.269)
	No	0.65		1.229(0.499 -3.026)
Anti-platelet drug	Yes	0.34	0.12	0.329(0.034 -3.182)
	No	0.10		2.076(0.880 -4.897)
OAC	WF	0.19	0.43	2.246(0.676 -7.461)
	DOAC	0.64		1.277(0.459 -3.551)
History of device implantation	Yes	N/A	N/A	none
	No	0.21		1.665 (0.756 -3.670)
Overall		0.25		1.577(0.732 -3.397)

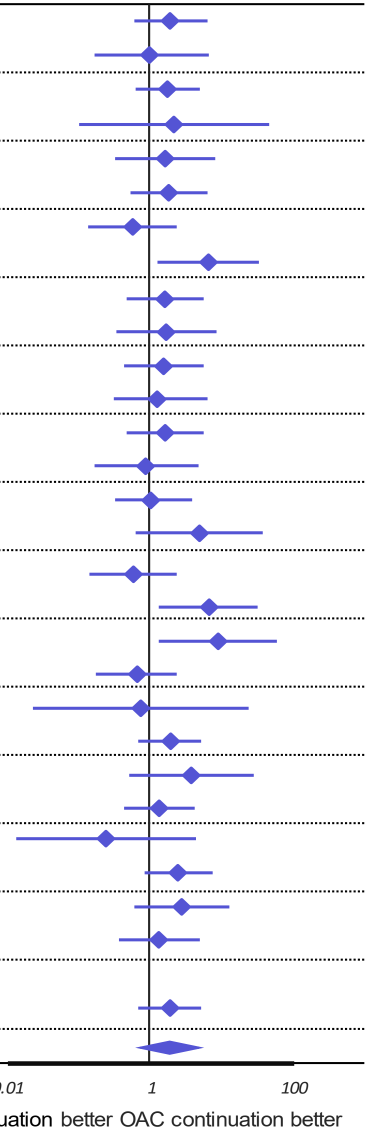

© 2025 Iwawaki T et al. *JAMA Network Open*.

B. Major bleeding events after PS-matched analysis

Variable		*P-value*	*P for interaction*	Hazard Ratio(95%CI)
Age	≥ 65	0.05	0.74	0.319(0.101 -1.004)
	< 65	0.15		0.209(0.025 -1.737)
Sex	male	0.23	0.24	0.489(0.151 -1.589)
	female	0.04		0.106(0.013 -0.853)
Body mass index	≥ 25	N/A	N/A	none
	< 25	0.27		0.543(0.185 -1.590)
Symptomatic AF	Yes	0.02	0.41	0.236(0.067 -0.827)
	No	0.55		0.594(0.109 -3.248)
Paroxysmal AF	Yes	0.08	0.97	0.310(0.085 -1.129)
	No	0.15		0.317(0.066 -1.527)
$CHADS_2$score	≥ 2	N/A	N/A	none
	< 2	0.16		0.474(0.167 -1.346)
CHA_2DS_2-VASc score	≥ 3	0.03	0.13	0.094(0.012 -0.753)
	< 3	0.32		0.550(0.169 -1.787)
HAS-BLED score	≥ 2	0.007	0.006	0.061(0.008 -0.461)
	< 2	0.30		2.440(0.447 -13.325)
LVEF	≥ 60	0.01	0.11	0.150(0.034 -0.665)
	< 60	0.90		0.911(0.203 -4.086)
LAD	≥ 45	0.74	0.55	0.662(0.060 -7.306)
	< 45	0.02		0.267(0.089 -0.805)
History of ischemic stroke	Yes	N/A	N/A	none
	No	0.02		0.314(0.116 -0.851)
History of HF	Yes	N/A	N/A	none
	No	0.05		0.359(0.131 -0.988)
Anti-platelet drug	Yes	0.02	0.10	0.083(0.011 -0.646)
	No	0.43		0.608(0.178 -2.082)
OAC	WF	0.04	0.22	0.117(0.015 -0.921)
	DOAC	0.28		0.516(0.155 -1.717)
History of device implantation	Yes	N/A	N/A	none
	No	0.04		0.339(0.123 -0.932)
Overall		0.02		0.310(0.114 -0.840)

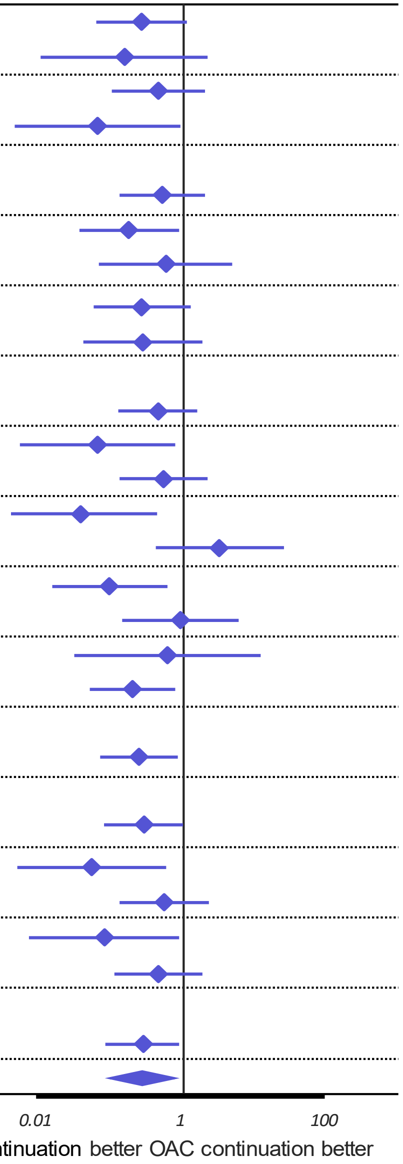

© 2025 Iwawaki T et al. *JAMA Network Open*.

C. All-cause death after PS-matched analysis

Variable		*P-value*	*P for interaction*	Hazard Ratio(95%CI)
Age	≥ 65	0.21	0.93	0.640(0.318 -1.288)
	< 65	0.55		0.692(0.208 -2.298)
Sex	male	0.23	0.58	0.647(0.316 -1.324)
	female	0.93		0.948(0.305 -2.947)
Body mass index	≥ 25	0.51	0.93	0.700(0.241 -2.031)
	< 25	0.45		0.754(0.363 -1.567)
Symptomatic AF	Yes	0.24	0.53	0.640(0.302 -1.356)
	No	0.95		0.966(0.350 -2.669)
Paroxysmal AF	Yes	0.36	0.91	0.718(0.354 -1.455)
	No	0.66		0.770(0.244 -2.429)
$CHADS_2$ score	≥ 2	0.99	0.44	0.991(0.368 -2.665)
	< 2	0.15		0.558(0.252 -1.234)
CHA_2DS_2-VASc score	≥ 3	0.29	0.74	0.597(0.230 -1.550)
	< 3	0.42		0.727(0.333 -1.588)
HAS-BLED score	≥ 2	0.09	0.24	0.512(0.234 -1.119)
	< 2	0.90		1.063(0.410 -2.757)
LVEF	≥ 60	0.44	0.99	0.727(0.323 -1.637)
	< 60	0.46		0.714(0.290 -1.755)
LAD	≥ 45	0.22	0.33	0.369(0.074 -1.841)
	< 45	0.59		0.836(0.433 -1.614)
History of ischemic stroke	Yes	N/A	N/A	none
	No	0.25		0.699(0.379 -1.289)
History of HF	Yes	0.03	0.02	0.233(0.064 -0.843)
	No	0.78		1.110(0.542 -2.272)
Anti-platelet drug	Yes	N/A	N/A	none
	No	0.76		0.907(0.483 -1.703)
OAC	WF	0.14	0.30	0.512(0.209 -1.257)
	DOAC	0.97		0.985(0.427 -2.274)
History of device implantation	Yes	0.82	0.98	0.774(0.090 -6.651)
	No	0.54		0.818(0.431 -1.550)
Overall		0.30		0.728(0.399 -1.328)

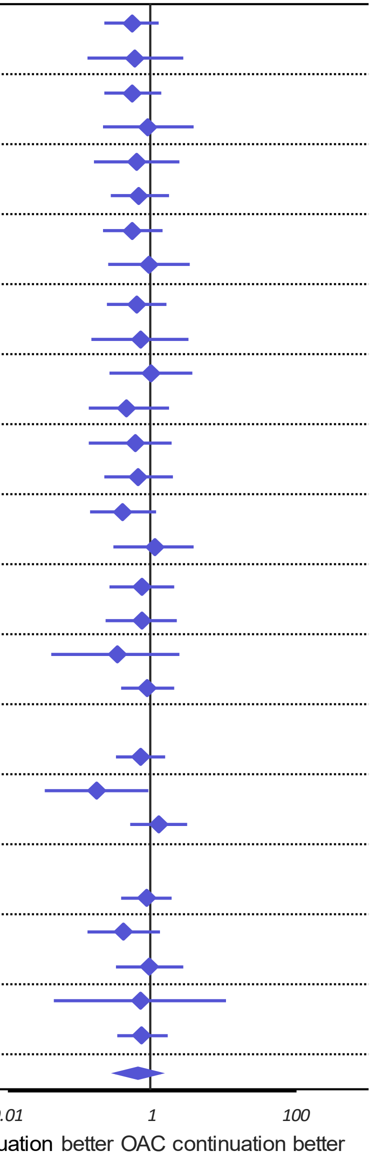

© 2025 Iwawaki T et al. *JAMA Network Open*.

OAC continuation was used as a reference.

AF, atrial fibrillation; CI, confidence interval; DOAC, direct oral anticoagulant; HF, heart failure; LAD, left atrial diameter; LVEF, left ventricular ejection fraction ;OAC, oral anticoagulant; PS, propensity score; WF, warfarin.

© 2025 Iwawaki T et al. *JAMA Network Open*.

eFigure 6. Kaplan-Meier Curve Analysis of Thromboembolic Events (A), Major Bleeding Events (B), and All-Cause Death (C) Between the OAC Discontinuation and Continuation Groups in the As-Treated Analysis

A. Thromboembolic events

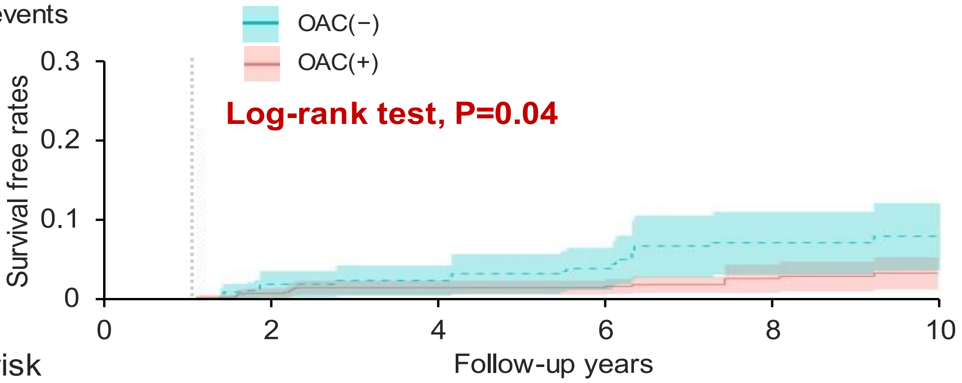

No. at risk

OAC(−)	916	529	385	268	175	111
OAC(+)	905	651	464	332	227	140

B. Major bleeding events

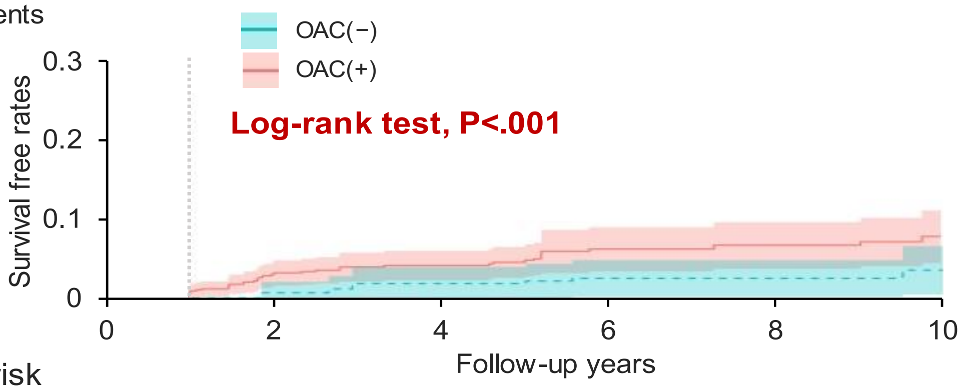

No. at risk

OAC(−)	916	532	387	267	177	112
OAC(+)	905	644	459	327	228	142

C. All-cause death

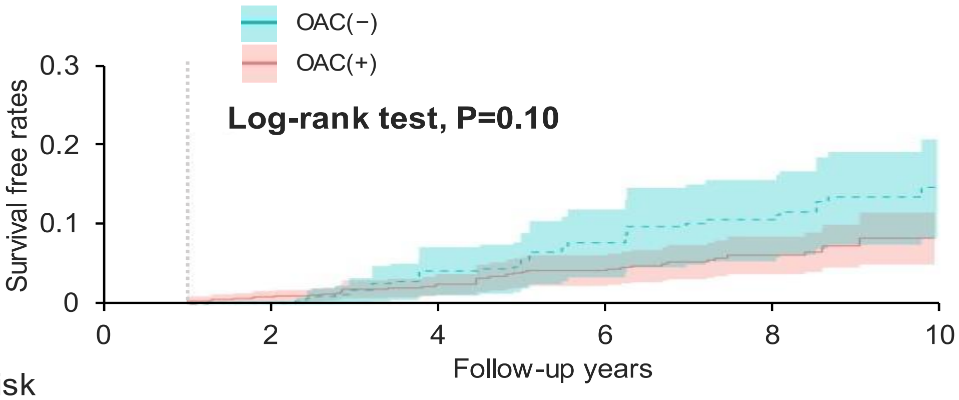

No. at risk

OAC(−)	916	533	389	271	180	114
OAC(+)	905	656	471	339	234	145

OAC, oral anticoagulant.

© 2025 Iwawaki T et al. *JAMA Network Open*.

eFigure 7. Kaplan-Meier Curve Analysis of Thromboembolic Events (A), Major Bleeding Events (B), and All-Cause Death (C) Between the OAC Discontinuation (N = 651) and Continuation (N = 1404) Groups After IPTW Analysis With a Landmark Period of 6 Months

A. Thromboembolic events

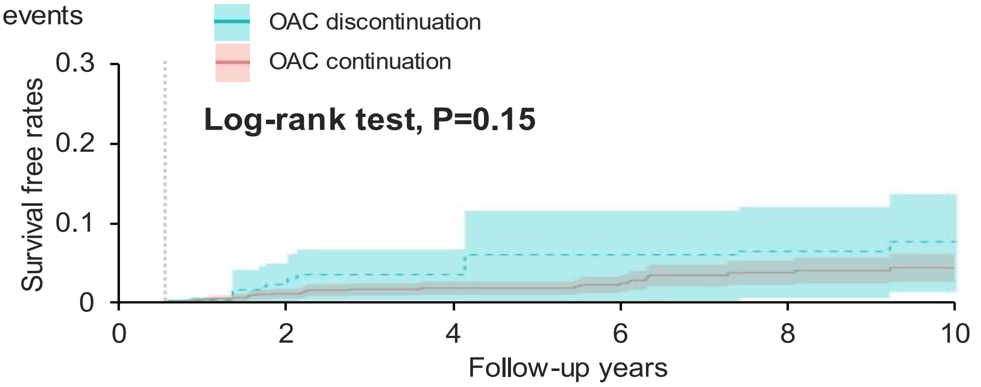

No. at risk

OAC discontinuation	651	339	239	178	122	75
OAC continuation	1404	917	659	457	308	186

B. Major bleeding events

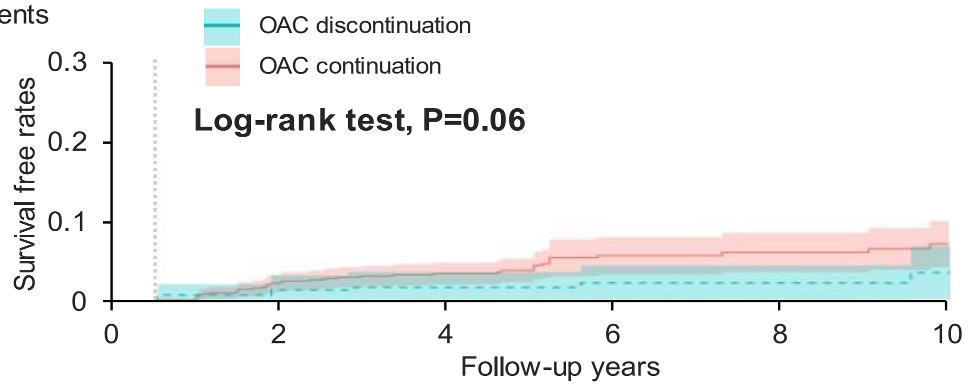

No. at risk

OAC discontinuation	651	340	241	178	121	75
OAC continuation	1404	913	654	452	312	190

C. All-cause death

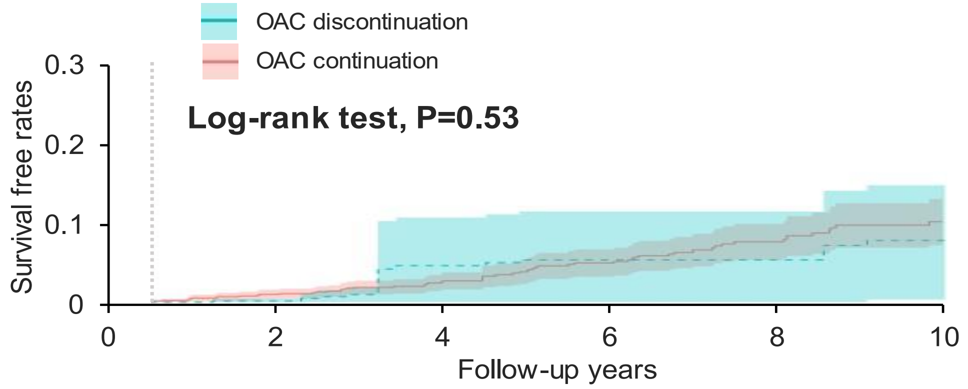

No. at risk

OAC discontinuation	651	342	243	181	124	77
OAC continuation	1404	925	668	466	319	194

IPTW, inverse probability of treatment weighting; OAC, oral anticoagulant.

© 2025 Iwawaki T et al. *JAMA Network Open*.

eReferences

1. Gage BF, Waterman AD, Shannon W, Boechler M, Rich MW, Radford MJ. Validation of clinical classification schemes for predicting stroke: results from the National Registry of Atrial Fibrillation. *JAMA*. Jun 13 2001;285(22):2864-70. doi:10.1001/jama.285.22.2864

2. Lip GY, Nieuwlaat R, Pisters R, Lane DA, Crijns HJ. Refining clinical risk stratification for predicting stroke and thromboembolism in atrial fibrillation using a novel risk factor-based approach: the euro heart survey on atrial fibrillation. *Chest*. Feb 2010;137(2):263-72. doi:10.1378/chest.09-1584

3. Lip GY, Frison L, Halperin JL, Lane DA. Comparative validation of a novel risk score for predicting bleeding risk in anticoagulated patients with atrial fibrillation: the HAS-BLED (Hypertension, Abnormal Renal/Liver Function, Stroke, Bleeding History or Predisposition, Labile INR, Elderly, Drugs/Alcohol Concomitantly) score. *J Am Coll Cardiol*. Jan 11 2011;57(2):173-80. doi:10.1016/j.jacc.2010.09.024

4. Yanagisawa S, Inden Y, Ohguchi S, et al. Periprocedural Management of Cardiac Tamponade During Catheter Ablation for AF Under Uninterrupted DOAC and Warfarin. *JACC Clin Electrophysiol*. Jul 2020;6(7):786-795. doi:10.1016/j.jacep.2020.02.005

5. Yanagisawa S, Inden Y, Fujii A, et al. Uninterrupted Direct Oral Anticoagulant and Warfarin Administration in Elderly Patients Undergoing Catheter Ablation for Atrial Fibrillation: A Comparison With Younger Patients. *JACC Clin Electrophysiol*. May 2018;4(5):592-600. doi:10.1016/j.jacep.2018.02.013

6. Schulman S, Kearon C. Definition of major bleeding in clinical investigations of antihemostatic medicinal products in non-surgical patients. *J Thromb Haemost*. Apr 2005;3(4):692-4. doi:10.1111/j.1538-7836.2005.01204.x

© 2025 Iwawaki T et al. *JAMA Network Open*.
