# Supplementary material for: Discontinuation of Oral Anticoagulation After Successful Atrial Fibrillation Ablation
Source: JAMA Netw Open. 2025 Mar 21;8(3):e251320. doi: 10.1001/jamanetworkopen.2025.1320 (PMC11929034; doi:10.1001/jamanetworkopen.2025.1320)
Supplement: Supplement 2. — Data Sharing Statement [file jamanetwopen-e251320-s002.pdf]

Data Sharing Statement

Iwawaki. Discontinuation of Oral Anticoagulation After Successful Atrial Fibrillation Ablation. *JAMA Netw Open*. Published March 21, 2025. doi:10.1001/jamanetworkopen.2025.1320

Data
Data available: No

Additional Information
Explanation for why data not available: Data was obtained retrospectively after IRB approval with a waiver of informed consent. As patients did not consent for the data to be shared, we cannot share the data.
